# Interplay Between J‐ and H‐Type Coupling in Aggregates of π‐Conjugated Polymers: A Single‐Molecule Perspective

**DOI:** 10.1002/anie.201912374

**Published:** 2019-11-06

**Authors:** Theresa Eder, Jan Vogelsang, Sebastian Bange, Klaas Remmerssen, Daniela Schmitz, Stefan‐S. Jester, Tristan J. Keller, Sigurd Höger, John M. Lupton

**Affiliations:** ^1^ Institut für Angewandte und Experimentelle Physik Universität Regensburg Universitätsstrasse 31 93053 Regensburg Germany; ^2^ Kekulé-Institut für Organische Chemie und Biochemie Universität Bonn Gerhard-Domagk-Strasse 1 53121 Bonn Germany

**Keywords:** electronic coupling, macrocycles, organic electronics, single-molecule spectroscopy

## Abstract

Strong dipole–dipole coupling within and between π‐conjugated segments shifts electronic transitions, and modifies vibronic coupling and excited‐state lifetimes. Since J‐type coupling between monomers along the conjugated‐polymer (CP) chain and H‐type coupling of chromophores between chains of a CP compete, a superposition of the spectral modifications arising from each type of coupling emerges, making the two couplings hard to discern in the ensemble. We introduce a single‐molecule H‐type aggregate of fixed spacing and variable length of up to 10 nm. HJ‐type aggregate formation is visualized intuitively in the scatter of single‐molecule spectra.

## Graphical abstract

**Coupling therapy**: In organic electronics, J‐type coupling between monomers along the chain and H‐type coupling of chromophores between chains compete, which makes the two types of coupling hard to discern in the ensemble. A single‐molecule H‐type aggregate of fixed spacing and variable length of up to 10 nm is introduced, and HJ‐type aggregate formation is visualized intuitively in the scatter of single‐molecule spectra.

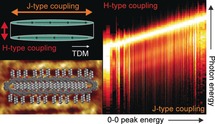

The term molecular—or organic—electronics is somewhat of an oxymoron. Molecules are, by their very definition, discrete entities, whereas the notion of electronics implies delocalization in describing the flow of electrons. Even if electronic wave functions delocalize within a molecule, how do charge and excitation energy pass from one molecule to the next within a solid? Whereas shape, electronic structure, and dynamics of even large molecules with delocalized π‐electron systems can, in principle, be rationalized from first principles, the nature of interactions of molecules within a solid remains a complex issue.^1^ Since extended π‐electron systems are highly polarizable, models of intermolecular dipole–dipole coupling—originally formulated to describe van‐der‐Waals‐bonded aggregates of dye molecules^2^—have been developed to explain the emergence of delocalized excitations within and between large π‐conjugated molecules such as conjugated polymers.^3^ The monomers of a polymer can be thought of as coupling to each other, adding up transition‐dipole moments (TDMs),3a, 4 to form a delocalized excited state resembling Jelley's original aggregates^5^ as sketched in Figure 1 a. A transition red‐shifted with respect to the monomer arises, accompanied by spectral narrowing due to reduced disorder and increased oscillator strength. The transition exhibits an increased radiative rate^6^ and a decrease in the relative vibrational coupling, which is characteristic of a delocalized state with a strongly allowed TDM.3f In contrast to this J‐type coupling, the interaction between chains aligned in parallel gives rise to an H‐aggregate, with a hypsochromic (blue‐)shift of the absorption.^7^ Transitions from the lower‐energy level of the split excited state become dipole‐forbidden,^8^ in effect because the individual TDMs of the co‐parallel molecular segments cancel out. Whereas a J‐aggregate can be viewed as arising from constructive interference of the individual TDMs, H‐aggregation constitutes destructive interference between a dipole and its image induced in the opposing segment. The degree of suppression of this collective TDM can be mitigated by molecular distortions.3g Such effects are often observed in conjugated polymers3b, 9 and are particularly pronounced on the single‐molecule level.^4^, ^10^

**Figure 1:**
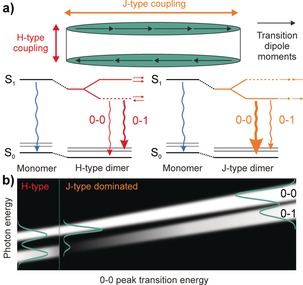
J‐ and H‐type coupling in π‐conjugated molecular dimers. a) Transition‐dipole moments (TDMs) of individual repeat units add to a J‐type aggregate excitation. The more ordered the chain, the greater the red‐shift and the weaker the relative vibrational coupling. H‐aggregation results from interactions between neighboring chromophores and suppresses radiative recombination, enhancing the ratio of vibronic‐to‐electronic fluorescence intensity. b) Anticipated correlation between the emission spectrum and the spectral shift, characterized by the 0–0 peak‐transition energy for single molecules. The relative electronic/vibronic emission intensities are shown, that is, the spectra are normalized to the electronic peak emission intensity.

Despite the interest in employing such models of electronic coupling to describe ensemble optical properties of complex molecules,3g few links of microscopic theory to microscopic experiments exist. In any ensemble, static and dynamic disorder^11^ control the interaction distances^12^ and the degree of electronic resonance^13^ between molecules, amplifying the effect of disorder broadening, which masks electronic coherences even in ultrafast experiments.^14^ Single‐molecule techniques have emerged as a route to study aggregation.^15^ With methods of controlled solvent‐vapor annealing, a few single molecules can be assembled to one single aggregate, probing the realm of single‐molecular mesoscopic structures with minimal disorder.^15^ Alternatively, multichromophoric covalently bound aggregates can be synthesized in which chromophores have a well‐defined orientation and spacing with respect to each other. Early work focused on perylene dimers, which showed signatures of either H‐ or J‐type coupling in the fluorescence lifetime and spectrum.^16^ More recently, larger model systems based on the chromophore segments of conjugated polymers have gained interest. We reported the effect of spacing in chromophore dimers^12^ and trimers^17^ on H‐type coupling, and were able to relate the scatter of the coupling strength on the single‐molecule level to quantum‐chemical calculations parameterized by molecular‐dynamics simulations.^17^ Microscopic experimental access to the interplay between H‐ and J‐type coupling at a level appropriate to theory necessitates varying both chain spacing and the effective chain length. As chain spacing decreases, the strength of H‐type coupling will increase.^12^ For the most strongly interacting co‐facial chains with most H‐character, intrachain order should also be highest: intrachain J‐aggregation will coincide with interchain H‐aggregation.^11^, ^18^ However, the two processes compete with each other. First, the longer the individual chain segment, the greater the potential degree of J‐type coupling.^4^, ^10^ This coupling will raise the radiative rate,^6^ reducing excited‐state interactions with the neighboring chain. Second, because of distortion of the molecular framework by excited‐state formation,^19^ localization will occur on dimensions much shorter than the chain. The longer the chain, the greater the possible separation between the localized states on opposing chains—which leads to weaker overall H‐type coupling.

Three metrics probe the interplay between intra‐ and interchain coupling: the spectral shift of the photoluminescence (PL) spectrum, which can be characterized by examining the 0–0 peak energy *E*_0–0_, the ratio of the vibronic‐to‐electronic luminescence intensity *I*_0–1_/*I*_0–0_, and the PL lifetime *τ*_PL_. Previously, we demonstrated a correlation between *E*_0–0_ and *τ*_PL_ on the single‐molecule level.^12^, ^17^ The same correlation is discussed for the molecules used in this work in Figure S15 (Supporting Information). However, since H‐ and J‐type coupling compete in their impact on spectroscopic observables, such a correlation on its own is not wholly satisfactory. Furthermore, it is not straightforward to discern radiative from non‐radiative contributions to *τ*_PL_, which may depend on the molecular conformation,^20^ the immediate dielectric environment,^21^ or intermolecular interactions.^12^, ^22^ Instead, we turn to an alternative analysis technique derived from another class of mesoscopic quantum emitters, semiconductor nanocrystals, where the sorting of many single‐particle spectra by *E*_0–0_ revealed different spectroscopic observables related to the quantum‐confined Stark effect.^23^ We apply this spectral correlation technique, sorting the single‐molecule spectra by their transition energy *E*_0–0_, to effectively reveal the individual constituents of the ensemble spectrum. The distribution of spectra arises because of conformational variability both along the individual chains of the dimer and between the chains. The effects of J‐ and H‐type aggregation compete and depend on chain conformation. Figure 1 b illustrates this approach schematically: the red‐most PL spectra arise from strong H‐aggregation and show the strongest vibronic intensity.3b, 3g, 24 However, strong H‐aggregation also implies strong J‐type coupling within the individual chains, the signatures of which are masked in the PL of the most strongly coupled H‐aggregate. Crucial information is apparent from the spectral trends. As the H‐type coupling strength decreases, the spectrum shifts to the blue and the relative intensity of the 0–1 transition suddenly drops. The individual chromophores of the dimer are dominated by J‐type coupling effects: the 0–1/0–0 peak‐intensity ratio increases continually as intrachain coupling diminishes and the PL shifts further to the blue. We expect that the strongest blue‐shifts arise due to torsional disorder on the chain, limiting the conjugation length.^25^ The shorter the conjugated part of the chain, the stronger the relative vibrational coupling.^26^

To test the interplay between intrachain J‐type coupling and inter‐chain H‐type coupling, we designed dimers of oligo(phenylene‐butadiynylene), a model system for studying intermolecular interactions.^27^ The conjugated units are 6 or 12 benzene rings long and spaced, on average, 4.6 Å apart by a biphenylene unit. Figure 2 shows the structure of the 12‐ring oligomer **1**, which we compare to the 6‐ring dimer **3** and the 12‐ring dimer **2**. High‐resolution scanning tunneling microscopy (STM) images on highly oriented pyrolytic graphite (Figure S7) illustrate how the sidechains of the structures interdigitate between the molecules. This perfect extension of the molecules will, however, not be given in the subsequent single‐molecule analysis, where the molecules are dispersed in a polystyrene host matrix and retain some residual flexibility.^17^ The Supporting Information gives details on the synthesis and characterization. In agreement with shorter dimer structures and related conjugated polymers,^28^ the three compounds show only small differences in absorption‐ and emission‐spectral features and *τ*_PL_ in the ensemble, as summarized in Figures S9 and S14. Significant differences in *τ*_PL_ are seen in the single‐molecule statistics (Figure S13) and in the correlation of *τ*_PL_ with the peak energy (Figure S15). **2** and **3** also show near‐perfect photon antibunching (Figure S10), implying that even though two chromophores are present, only one excited state emits at a time. Additionally, on the single‐molecule level, almost all dimers adopt an extended conformation since they show a high degree of excitation‐polarization anisotropy in their emission as the polarization plane of the exciting laser is rotated (Figure S11).

**Figure 2:**
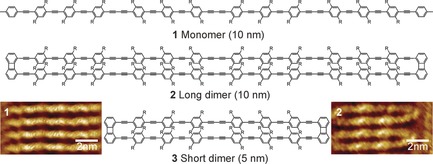
Chemical structures of the model HJ‐aggregates with STM images of samples **1** and **2** on a graphite surface (11×5.5 nm^2^ image size).

We begin by discussing the PL spectra of single molecules of the oligomer **1**. As described in the Supporting Information, we measured spectra for single molecules dispersed in a spin‐coated polystyrene (PS) matrix, excited at 405 nm under ambient conditions to minimize triplet build‐up and thus photoquenching. Examples of individual single‐molecule spectra are shown in Figure S12. Data analysis and spectral fitting procedures are explained in Figure S8. Figure 3 a–c summarizes the single‐molecule data for the monomer **1**, ordering the spectra by their 0–0 peak energy *E*_0–0_. The spectra show a clear trend of diminishing 0–1/0–0 intensity ratio with decreasing E_0–0_, characteristic of a J‐aggregate. Although it is well known that single‐molecule PL spectra scatter in energy due to the different molecular conformations and dielectric environments probed,^20^, ^29^ such a clear correlation between distinct spectroscopic observables as *E*_0–0_ and the vibronic intensity ratio *I*_0–1_/*I*_0–0_, as in panel (b), is rarely identified. The green lines indicate averaging over 50 points. Following Knapp,^30^ increasing the number of TDMs coupled in‐line decreases the spectral linewidth. This effect is seen in panel (c) in terms of the full width at half maximum (FWHM) of the 0–0 peak as a function of *E*_0–0_.

**Figure 3:**
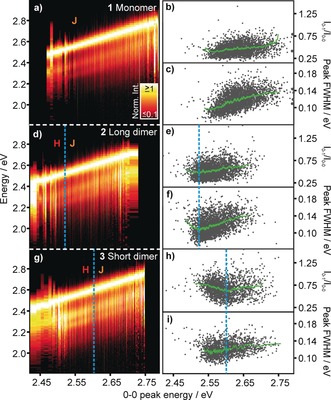
PL spectra of 2875, 2743, and 1857 single molecules of **1**, **2** and **3**, sorted by the 0–0 peak‐transition energy *E*_0–0_ and normalized to *I*_0–0_. In **1**, the vibronic‐intensity ratio *I*_0–1_/*I*_0–0_ and spectral FWHM decreases with decreasing *E*_0–0_, as expected for J‐aggregation. In **2** and **3**, spectral signatures of J‐aggregation within the chromophores are identified in analogy to **1**. Above a certain red‐shift, the spectral shape changes and H‐aggregation dominates: the spectra broaden and the oscillator strength shifts to the 0–1 transition. This threshold is indicated in blue. The vibronic‐intensity ratio increases on either side of the set threshold. Green lines indicate averages over 50 points.

Figure 3 d–f shows the correlation plot for the long dimer **2** along with the vibronic intensity ratio and FWHM as a function of *E*_0–0_. We discern the J‐type coupling of the monomer units from the H‐type coupling of the dimer by the redistribution of the oscillator strength to the vibronic transition for the most red‐shifted emission: starting from the lowest *E*_0–0_ and moving higher, the intensity of the vibronic sideband first decreases and then increases again, corresponding to a suppression of H‐type coupling followed by a decrease in J‐type coupling. The same behavior is seen more clearly in the correlation plot of the peak FWHM in panel (f). While the H‐type coupling is only weakly visible for the long dimer, it should become more pronounced for the short dimer as stated above. Qualitatively, single‐molecule spectra of the short dimer **3** in panels Figure 3 g–i also follow this trend, although the J‐type character is less pronounced. The H‐type coupling is clearly visible as a distinctive increase of *I*_0–1_/*I*_0–0_ at decreasing *E*_0–0_ values. A similar correlation is also seen in the Huang–Rhys factors of the spectra, as discussed in Figure S16. This representation of large sets of data offers an intuitive visualization of the non‐trivial interplay between J‐ and H‐type coupling in multichromophoric aggregates. In particular, the transition from J‐ to H‐type behavior, indicated by the dashed blue lines, conveys the impression that a quantitative analysis of dipole‐coupling strengths could be possible. We note that the overall PL intensity does not decrease significantly in H‐type aggregates. The radiative rate decreases in such aggregates, but the PL quantum yield remains almost the same due to a negligible overall non‐radiative rate in these molecules.^12^ A complete analysis of these characteristics would require careful considerations of the microscopic molecular dynamics involved, since the backbone can be quite flexible and the strongest H‐type couplings may occur only within a small region of the overall segment.^17^

The noise in the scatter plots of the peak width and the *I*_0–1_/*I*_0–0_ ratio in Figure 3 should also be noted. The data for **2** and **3** show a somewhat larger scatter with the transition energy than for **1**. The reason for this difference is simple: the additional degree of freedom—interchromophoric spacing, which determines the strength of H‐type coupling in **2** and **3**—does not necessarily correlate fully with the effective chromophore length, that is, with the degree of J‐type coupling. Very small variations in the interchromophoric spacing, as predicted by molecular‐dynamics simulations,^17^ have a drastic impact on the spectral width and vibronic coupling without necessarily affecting the transition energy, which is dominated by the degree of J‐type coupling. These fluctuations show up as noise in the 2D plots, since, even in the regime where the spectra are dominated by J‐coupling, H‐type interactions can still occur, giving rise to spectral broadening and a suppression of *I*_0–0_. Finally, we stress that this analysis approach is universal and can be applied to a range of different molecules. Three further examples of dimers and a trimer are summarized in Figure S17, giving correlation plots analogous to those of Figure 3.

Single‐molecule spectroscopy is the experiment most closely related to microscopic theory, but it is often challenging to analyze and represent data spanning the ensemble heterogeneity in a statistically meaningful way. Here, we illustrate an intuitive visualization of the subtle interplay between J‐ and H‐type coupling, which is inherent to any ensemble of π‐conjugated materials. The method is readily portable to different conjugated backbones, provided that suitable dimers can be synthesized. In particular, it is expected to be highly sensitive to the influence of backbone substituents, which have a drastic impact on the bulk packing.^15^ We also expect the competition between intrachain J‐type and interchain H‐type aggregation to give rise to non‐trivial phenomena at low temperatures, where aggregation effects in the spectra should be enhanced due to a decrease in dynamic disorder: the J‐character should become even more pronounced and should show signatures of superradiance,^6^, ^16^ whereas vibrational perturbation of the excited state and hence luminescence from the H‐aggregate will become suppressed as molecular dynamics are tempered.8b

## Conflict of interest

The authors declare no conflict of interest.

## Supporting information

As a service to our authors and readers, this journal provides supporting information supplied by the authors. Such materials are peer reviewed and may be re‐organized for online delivery, but are not copy‐edited or typeset. Technical support issues arising from supporting information (other than missing files) should be addressed to the authors.

Supplementary
