# Supplementary material for: Interplay Between J‐ and H‐Type Coupling in Aggregates of π‐Conjugated Polymers: A Single‐Molecule Perspective
Source: Angew Chem Int Ed Engl. 2019 Nov 6;58(52):18898–902. doi: 10.1002/anie.201912374 (PMC6973276; doi:10.1002/anie.201912374)
Supplement: Supplementary file 1 — Supplementary [file ANIE-58-18898-s001.pdf]

Angewandte Chemie
Eine Zeitschrift der Gesellschaft Deutscher Chemiker

Supporting Information

Interplay Between J- and H-Type Coupling in Aggregates of π-Conjugated Polymers: A Single-Molecule Perspective

Theresa Eder, Jan Vogelsang, Sebastian Bange, Klaas Remmerssen, Daniela Schmitz, Stefan-S. Jester, Tristan J. Keller, Sigurd Höger,* and John M. Lupton**

anie_201912374_sm_miscellaneous_information.pdf

Table of contents **Page**

1. Details of Synthesis S2
   1.1 General information S2
   1.2 Compounds S3
   1.3 Synthetic strategy S3
   1.4 Synthesis S5
   1.5 Additional compounds S19
2. Scanning tunneling microscopy S27
3. Ensemble measurements S28
4. Single molecule sample preparation and setup S28
5. Spectral fitting procedure S30
6. Supporting Figures S31

1. Synthesis

1.1 General information

Reagents were purchased at reagent grade from commercial sources and used without further purification. All air-sensitive reactions were performed by applying standard Schlenk techniques under Ar atmosphere and – if not otherwise indicated – using dry solvents (THF, piperidine, diisopropylamine). The latter were dried, distilled and stored under Ar according to standard methods. Workup solvents were purified by distillation. All solids and oils were dried overnight at r.t. under vacuum prior to characterization and further processing. Microwave reactions were performed in a Discover Labmate microwave reactor (2450MHz) from CEM GmbH in 10 mL-pressure tubes filled in a glovebox under Ar. 1H and 13C NMR spectra were recorded on Bruker Avance I 400 MHz (1H: 400.1 MHz, 13C: 100.6 MHz), Bruker Avance I 500 MHz (1H: 500.1 MHz, 13C: 125.8 MHz), Bruker Avance III HD 500.1 MHz (1H: 500 MHz, 13C: 125.8 MHz), Bruker Avance III HD 700MHz (1H: 700.1 MHz, 13C: 176.0 MHz). Chemical shifts are given in parts per million (ppm) referenced to residual 1H or 13C signals in deuterated $CDCl_3$ (1H: 7.26, 13C: 77.0) and CD_2Cl_2 (1H: 5.32, 13C: 53.5). Deuterated solvents were obtained from Deutero GmbH, Germany. Mass spectra were measured on a Finnigan ThermoQuest MAT 95 XL (EI-MS), and a Bruker Daltronics autoflex II TOF/TOF (MALDI-MS; matrix material: DCTB, no salts added). Thin layer chromatography was performed on silica gel coated aluminium plates (Macherey-Nagel, Alugramm SIL G/UV254, 0.2 mm silica gel coating with fluorescence indicator). Column chromatography was performed using silica gel 60 M (Macherey-Nagel, 40-63 µm) as stationary phase. Gel permeation chromatography (GPC) was performed in THF (p.a. grade, stabilized with 2.5 ppm BHT) at r.t. For the separation of the compounds, a Shimadzu Recycling GPC system, equipped with a LC-20 AD pump, an SPD-20 A UV detector and a set of three preparative columns from PSS (Polymer Standards Service, Mainz, Germany, 103 Å, 5 µ, 20 mm $\times$ 300 mm) was employed. The system was operated at a flow rate of 6 mL/min.

1.2 Compounds

Figure S1 gives an overview of the oligomer **1** and the bichromophoric compounds **2** and **3**.

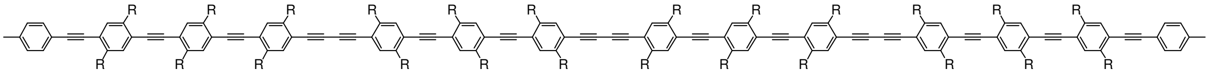

1, R = OC_6H_{13}

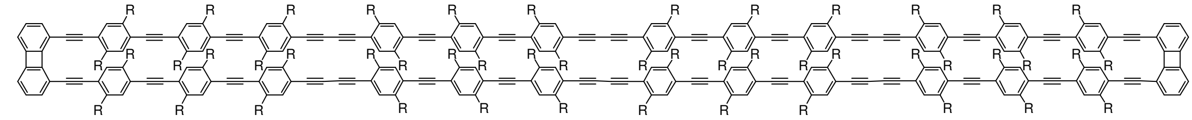

2, R = OC_6H_{13}

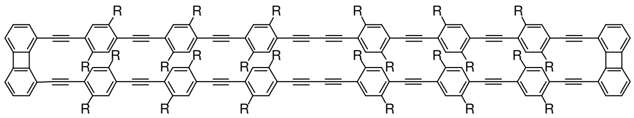

3, R = OC_6H_{13}

Figure S1. Structures of **1, 2** and **3**.

1.3 Synthetic strategy

Oligomer **1** is synthesized by Sonogashira coupling of the corresponding bisacetylene **4**[1] with 4-iodotoluene (**5**) (Scheme S1a).

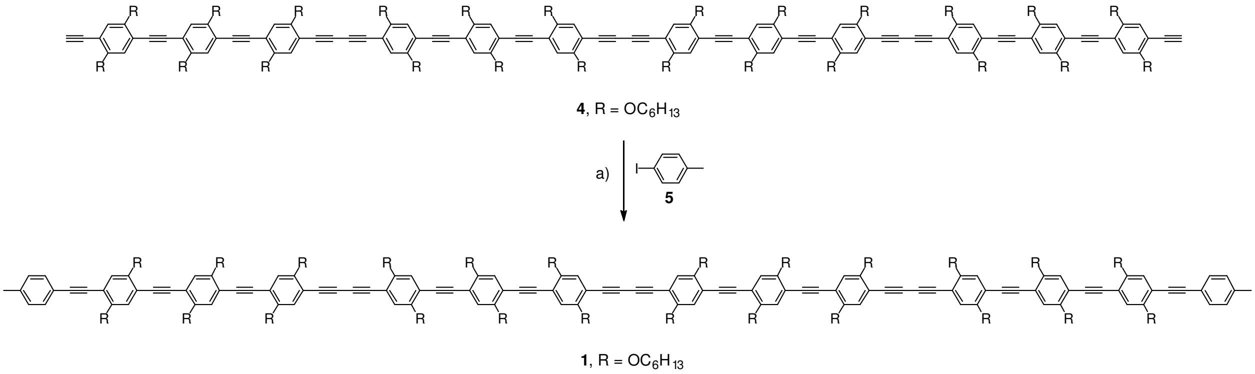

Scheme S1a. a) PdCl2(PPh3)2, CuI, PPh3, piperidine, THF, r.t., overnight, 36 %.

The bichromophoric macrocyles **2** and **3** are synthesized in an analogous reaction sequence as it is previously described for similar macrocycles.[1] Throughout the synthesis, as well as for the synthesis of **4**, **6a** and **6b**, the polar CPDIPS-protecting group[2] allows the purification of the intermediates by column chromatography over silica gel. This approach, in combination with the recGPC purification, guarantees the purity and monodispersity of all compounds described here. Sonogashira-Hagihara reaction of the mono-protected acetylene terminated oPEB **6a**/**6b** and the clamp unit **7** yields **8a**/**8b**. The subsequent statistical deprotection gives the mono-protected half ring **9a**/**9b** which is then dimerized. The macrocycle is then formed by cleavage of the

CPDIPS protecting groups, followed by an intramolecular ring closure under palladium catalysis at high dilution (Scheme S1b).

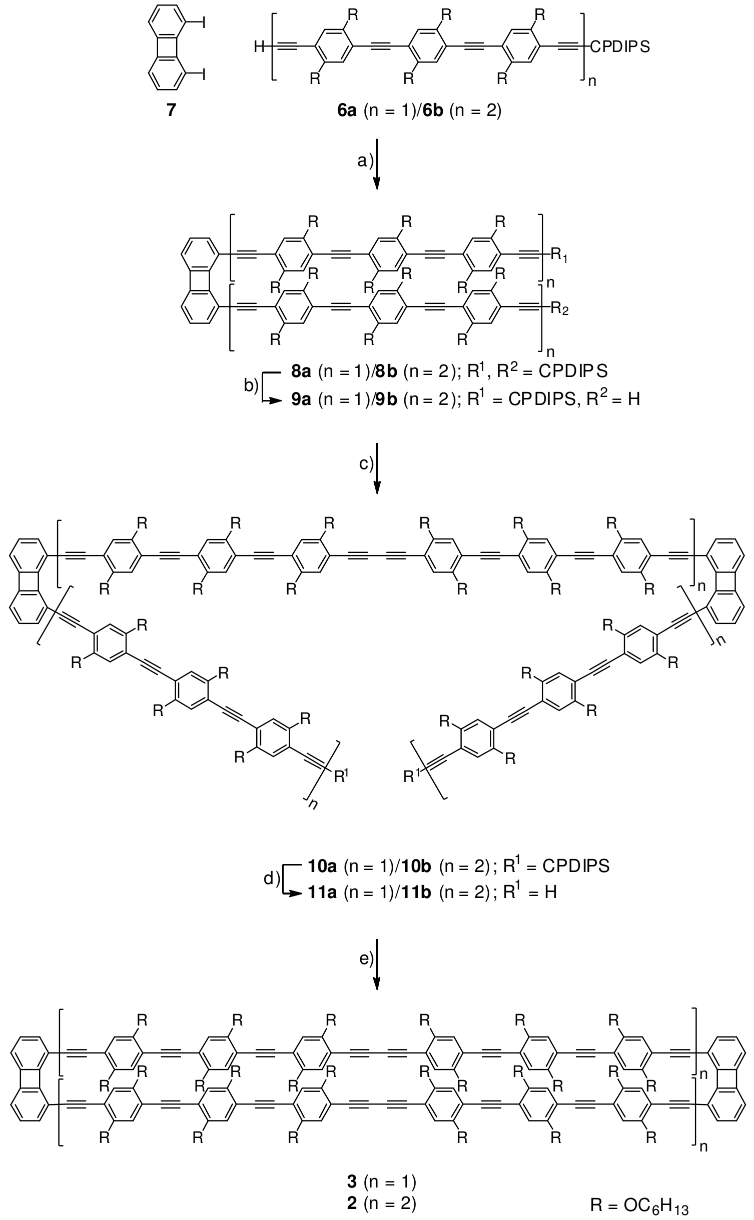

Scheme S1b. a) $PdCl_2(PPh_3)_2$, CuI, PPh_3, piperidine, THF, microwave, 120 °C, 30 min, 69 % (**8a**); 120 °C, 40 min, 48 % (**8b**). b) TBAF, THF, water, r.t., 2.5 h, 29 % (**9a**); r.t., 4.5 h, 29 % (**9b**). c) $PdCl_2(PPh_3)_2$, CuI, I_2, THF, diisopropylamine, r.t., 24 h, 90 % (**10a**); r.t., 16 h, 68 % (**10b**); d) TBAF, THF, r.t., 1.5 h, 87 % (**11a**); r.t., 16 h, 81 % (**11b**); e) $PdCl_2(PPh_3)_2$, CuI, I_2, THF, diisopropylamine, 40 °C, 40 h, 33 % (**2**); 40 °C, 71 h, 22 % (**3**).

1.4 Synthesis

1

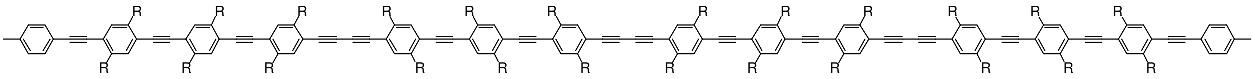

1, R = OC_6H_{13}

Under an argon atmosphere, **4**[1] (17 mg, 4.6 µmol) and **5** (10 mg, 46 µmol), $PdCl_2(PPh_3)_2$ (0.1 mg, 0.1 µmol), CuI (1 mg, 5 µmol) and PPh_3 (1 mg, 4 µmol) were dissolved in THF (3 mL) and piperidine (3 mL) and stirred over night at r.t. The reaction was diluted with water and dichloromethane. The aqueous phase was extracted with dichloromethane and the combined organic phase was washed with sulfuric acid (10 %) and water and brine, and dried over magnesium sulfate. After removal of the solvent, the crude product was purified by filtering through a plug of silica gel and subsequent recGPC to give **1** (7 mg, 1.7 µmol, 36 %) as a yellow solid.

^{1}H-NMR (500 MHz, CD_2Cl_2, RT) δ [ppm]: 7.42 (d, J = 8.0 Hz, 4 H), 7.19 (d, J = 8.0 Hz, 4 H), 7.03-7.01 (m, 24 H), 4.06-4.01 (m, 48 H), 2.38 (s, 6 H), 1.88-1.81 (m, 48 H), 1.56-1.49 (m, 48 H), 1.40-1.33 (m, 96 H), 0.94-0.88 (m, 72 H).

^{13}C-NMR (125.8 MHz, CD_2Cl_2, RT) δ [ppm]: 155.05, 153.57, 153.53, 153.49, 153.33, 138.84, 131.38, 129.22, 120.26, 117.66, 117.04, 116.97, 116.86, 116.78, 115.37, 115.32, 114.37, 114.25, 114.13, 113.89, 113.78, 112.19, 112.15, 95.05, 92.30, 92.24, 91.71, 91.47, 91.36, 85.36, 79.77, 78.95, 69.71, 69.68, 69.63, 31.67, 31.61, 29.40, 29.34, 29.27, 29.17, 25.81, 25.73, 25.70, 25.66, 22.72, 22.68, 21.30, 13.89.

MS (MALDI-pos, DCTB) $C_{262}H_{350}O_{24}$ (3880.62) m/z (%): 3883.6 (100) $[M]^+$, 4133.8 (85) $[M + DCTB]^+$, 4384.6 (30) $[M + 2 DCTB]^+$, 4634.1 (5) $[M + 3 DCTB]^+$.

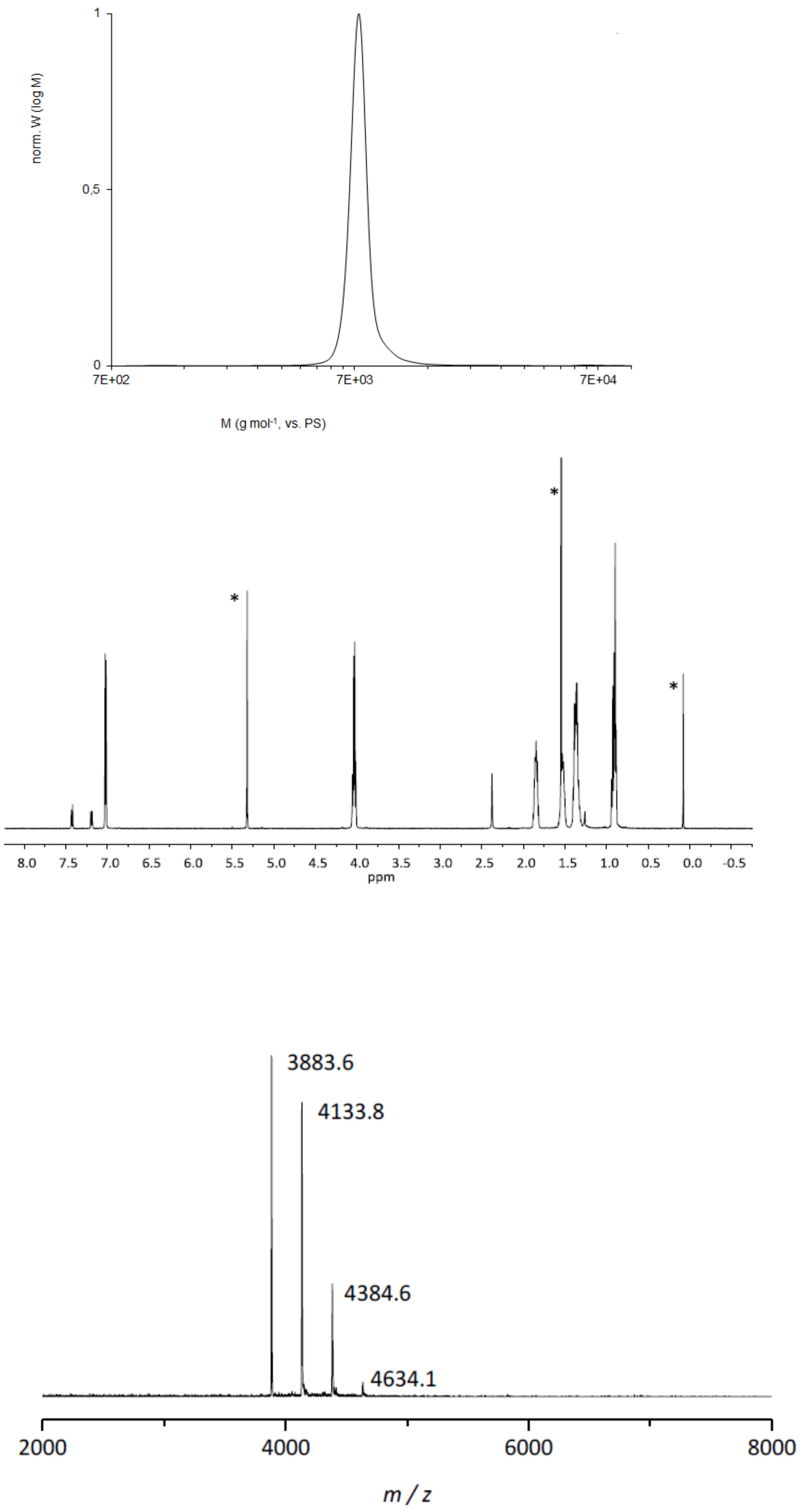

Figure S2: Analytical GPC, ^{1}H-NMR (CD_2Cl_2) spectrum and MALDI spectrum of **1** (from top to down).

8a

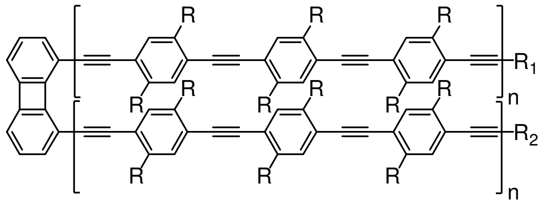

8a (n = 1) R^1, R^2 = CPDIPS, R = OC_6H_{13}

Under an argon atmosphere, **7**[3] (10 mg, 32 µmol) and **6a**[1] (66 mg, 60 µmol), $PdCl_2(PPh_3)_2$ (1 mg, 1 µmol), CuI (1 mg, 5 µmol) and PPh_3 (1 mg, 4 µmol) were placed in a microwave tube and THF (1.5 mL) and piperidine (1.5 mL) were added and the mixture was heated to 120 °C for 30 min. The reaction was diluted with water and dichloromethane. The aqueous phase was extracted with dichloromethane and the combined organic phase was washed with hydrochloric acid (2 M), water and brine and dried over magnesium sulfate. After removal of the solvent, the crude product was purified by column chromatography (cyclohexane:dichloromethane 2:3, R_f = 0.15) to give **8a** (44 mg, 18 µmol, 69 %) as a yellow film.

Formula: $C_{156}H_{214}N_2O_{12}Si_2$, molecular weight: 2365.6 g/mol.

^{1}H-NMR (500 MHz, $CDCl_3$, r.t.) δ [ppm]: 6.97 (s, 2 H), 6.96 (s, 2H), 6.94 (s, 2H), 6.92 (s, 2H), 6.92 (s, 2H), 6.90 (d, J = 8.4 Hz, 2H), 6.77 (dd, J = 6.8 Hz, J = 1.7 Hz, 2 H), 6.76 (s, 2 H), 6.60 (d, J = 6.8 Hz, 2 H), 4.06 - 3.92 (m, 16 H), 3.89 (t, J = 6.5 Hz, 4 H), 3.47 (t, J = 6.5 Hz, 4 H), 2.43 (t, J = 7.0 Hz, 4 H), 1.93 - 1.86 (m, 4 H), 1.85 - 1.74 (m, 24 H), 1.70 - 1.62 (m, 4 H), 1.57 - 1.44 (m, 24 H), 1.43 - 1.22 (m, 48 H), 1.16 - 1.05 (m, 28 H), 0.94 - 0.78 (m, 36H).

^{13}C-NMR (125.6 MHz, $CDCl_3$, r.t.) δ [ppm]: 154.43, 153.82, 153.63, 153.62, 153.47, 153.34, 151.43, 150.52, 132.24, 128.73, 119.90, 117.88, 117.26, 117.18, 116.80, 116.64, 116.61, 116.51, 115.02, 114.62, 114.50, 114.25, 114.18, 113.74, 113.31, 104.11, 95.18, 92.51, 92.02, 91.85, 91.77, 91.51, 90.04, 69.94, 69.71, 69.68, 69.48, 69.24, 69.18, 31.90, 31.86, 31.76, 31.73, 29.51, 29.45, 29.44, 29.42, 25.99, 25.92, 25.83, 25.81, 25.78, 22.81, 22.78, 22.76, 21.43, 20.90, 18.37, 18.13, 14.20, 14.19, 14.17, 14.16, 11.95, 9.78.

MS (MALDI-pos, DCTB) m/z = 2363.5 $[M]^+$, 2613.7 $[M + DCTB]^+$.

8b

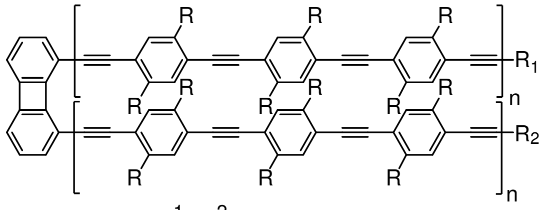

8b (n = 1) R^1, R^2 = CPDIPS, R = OC_6H_{13}

Under an argon atmosphere, **7**[3] (4.8 mg, 16 µmol) and **6b**[1] (67 mg, 33 µmol), $PdCl_2(PPh_3)_2$ (3 mg, 2.6 µmol), CuI (0.5 mg, 2.6 µmol) and PPh_3 (3 mg, 11 µmol) were placed in a microwave tube and THF (5 mL) and piperidine (2 mL) were added and the mixture was heated to 120 °C for 40 min. The reaction was diluted with water and dichloromethane. The aqueous phase was extracted with dichloromethane and the combined organic phase was washed with hydrochloric acid (2 M), water and brine and dried over magnesium sulfate. After removal of the solvent, the crude product was purified by column chromatography (cyclohexane:dichloromethane 1:1 to 1:3, R_f = 0.31 (1:3)) to give **8b** (32 mg, 7.5 µmol, 48 %) as a yellow-orange film.

Formula: $C_{280}H_{382}N_2O_{24}Si_2$, molecular weight: 4216.3 g/mol.

^{1}H-NMR (500 MHz, $CDCl_3$, r.t.) δ [ppm]: 7.04 - 6.88 (m, 24 H), 6.80 - 6.73 (m, 4 H), 6.60 (d, J = 6.8 Hz, 2 H), 4.11 - 3.93 (m, 40 H), 3.90 (t, J =6.5 Hz, 4 H), 3.47 (t, J =6.5 Hz, 4 H), 2.43 (t, J = 7.0 Hz, 4H), 1.94 - 1.74 (m, 48 H), 1.73 - 1.59 (m, 4 H), 1.58 - 1.45 (m, 48 H), 1.41 - 1.24 (m, 96 H), 1.18 - 1.06 (m, 28-H), 0.96 - 0.78 (m, 76 H).

^{13}C-NMR (125.8 MHz, $CDCl_3$, r.t.) δ [ppm]: 155.12, 154.46, 153.84, 153.70, 153.69, 153.64, 153.50, 153.47, 153.45, 153.39, 151.45, 150.54, 132.25, 128.75, 119.91, 117.97, 117.93, 117.38, 117.37, 117.31, 117.24, 117.20, 116.83, 116.65, 116.58, 115.65, 115.54, 114.94, 114.77, 114.52, 114.34, 114.27, 114.13, 113.79, 113.43, 112.74, 112.64, 108.07, 104.10, 95.27, 92.53, 92.49, 92.33, 92.09, 91.78, 91.72, 91.68, 91.54, 91.43, 90.06, 79.79, 79.72, 79.52, 79.46, 69.99, 69.90, 69.89, 69.84, 69.82, 69.81, 69.75, 69.73, 69.51, 69.29, 69.21, 67.82, 31.92, 31.89, 31.87, 31.78, 31.76, 31.75, 31.73, 31.69, 30.46, 29.84, 29.53, 29.47, 29.45, 29.44, 29.43, 29.36, 29.35, 29.27, 26.00, 25.93, 25.89, 25.84, 25.82, 25.81, 25.79, 25.78, 25.77, 24.05, 22.82, 22.81, 22.79, 22.78, 22.74, 22.64, 21.45, 20.91, 18.38, 18.14, 14.21, 14.19, 14.18, 14.17, 11.96, 9.80, 1.16.

MS (MALDI-pos, DCTB) m/z = 4216.3 $[M]^+$, 4466.4 $[M + DCTB]^+$.

9a

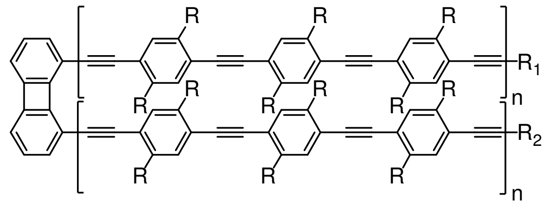

9a (n = 1) R^1 = CPDIPS, , R^2 = H, R = OC_6H_{13}

Under an argon atmosphere, **8a** (142 mg, 60 µmol) was dissolved in THF (4.5 mL) and water (0.22 mL). A 1 M solution of TBAF in THF (0.1 mL, 100 µmol) was added. The reaction was stirred and monitored by TLC. After 30 min, additional TBAF in THF (0.1 mL, 100 µmol) was added, 30 min later again (0.05 mL, 50 µmol), 30 min later again (0.05 mL, 50 µmol), and 30

min later again (0.25 mL, 250 µmol). After a total reaction time of 2.5 h the mixture was diluted with water and dichloromethane. The aqueous phase was extracted with dichloromethane and the combined organic phase was washed with water and brine and dried over magnesium sulfate. After removal of the solvent, the crude product was purified by column chromatography (cyclohexane:dichloromethane 1:1, R_f = 0.32 (2:3)) to give **9a** (38 mg, 17 µmol, 29 %) as a yellow solid.

Formula: $C_{146}H_{195}NO_{12}Si$, molecular weight: 2184.3 g/mol.

^{1}H-NMR (400 MHz, $CDCl_3$, r.t.) δ [ppm]: 6.98 - 6.95 (m, 6 H), 6.94 (s, 1 H), 6.92 - 6.88 (m, 5 H), 6.77 (dd, J =6.8 Hz, J =1.7 Hz, 2 H), 6.76 (s, 2 H), 6.60 (d, J = 6.8 Hz, 2 H), 4.04 - 3.91 (m, 16 H), 3.89 (t, J = 6.5 Hz, 4 H), 3.47 (t, J = 6.5 Hz, 4 H), 3.34 (s, 1 H), 2.43 (t, J = 7.0 Hz, 2 H), 1.94 - 1.72 (m, 22 H), 1.71 - 1.61 (m, 4 H), 1.55 - 1.43 (m, 22-H), 1.42 - 1.22 (m, 56-H), 1.15 - 1.09 (m, 14 H), 0.97 - 0.76 (m, 32 H).

^{13}C-NMR (125.8 MHz, $CDCl_3$, r.t.) δ [ppm]: 154.44, 154.27, 153.82, 153.66, 153.64, 153.61, 153.47, 153.42, 153.34, 151.46, 151.45, 150.53, 132.25, 128.74, 119.92, 118.04, 117.88, 117.28, 117.26, 117.20, 117.17, 117.14, 116.79, 116.65, 116.60, 116.50, 115.17, 115.01, 114.66, 114.62, 114.50, 114.26, 114.18, 114.14, 113.74, 113.31, 112.61, 104.11, 95.19, 92.50, 92.02, 91.85, 91.83, 91.78, 91.77, 91.52, 91.28, 90.04, 82.38, 80.21, 77.09, 69.94, 69.84, 69.73, 69.72, 69.70, 69.68, 69.48, 69.24, 69.18, 31.92, 31.87, 31.79, 31.78, 31.75, 31.73, 31.68, 29.53, 29.52, 29.46, 29.45, 29.43, 29.37, 29.28, 26.00, 25.95, 25.94, 25.83, 25.82, 25.79, 25.78, 25.75, 22.82, 22.79, 22.77, 22.73, 21.44, 20.92, 18.39, 18.14, 14.22, 14.20, 14.18, 14.17, 14.16, 11.95, 9.78.

MS (MALDI-pos, DCTB) m/z = 2182.3 $[M]^+$, 2432.4 $[M + DCTB]^+$.

9b

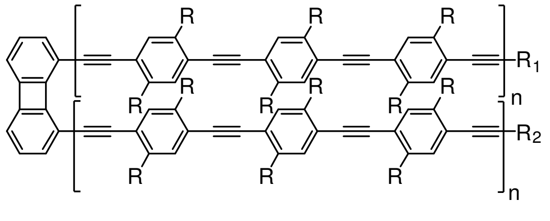

9b (n = 2) R^1 = CPDIPS, R^2 = H, R = OC_6H_{13}

Under an argon atmosphere, **8b** (73 mg, 17 µmol) was dissolved in THF (6 mL) and water (0.26 mL). A 1 M solution of TBAF in THF (0.04 mL, 40 µmol) was added. The reaction was stirred and monitored by TLC. After 1h additional TBAF in THF (0.04 mL, 40 µmol) was added, 1h later again (0.04 mL, 40 µmol), 1h later again (0.04 mL, 40 µmol), 1h later again (0.04 mL, 40 µmol) and 1h later again (0.04 mL, 40 µmol). After a total reaction time of 4.5 h the mixture

was diluted with water and dichloromethane. The aqueous phase was extracted with dichloromethane and the combined organic phase was washed with water and brine and dried over magnesium sulfate. After removal of the solvent, the crude product was purified by column chromatography (cyclohexane:dichloromethane 1:1 to 1:3, R_f = 0.44 (1:3)) to give **9b** (20 mg, 5 µmol, 29 %) as a yellow-orange film.

Formula: $C_{270}H_{363}NO_{24}Si$, molecular weight: 4034.9 g/mol.

^{1}H-NMR (700 MHz, $CDCl_3$, r.t.) δ [ppm]: 7.03 - 6.88 (m, 24 H), 6.80 - 6.74 (m, 4 H), 6.60 (d, J = 6.8 Hz, 2 H), 4.07 - 3.93 (m, 40 H), 3.90 (t, J =6.5 Hz, 4 H), 3.47 (t, J =6.5 Hz, 4 H), 3.34 (s, 1 H), 2.43 (t, J =7.0 Hz, 2 H), 1.95 - 1.74 (m, 48 H), 1.72 - 1.60 (m, 2 H), 1.55 - 1.43(m, 48 H), 1.41 - 1.24 (m, 96 H), 1.17 - 1.05 (m, 14 H), 0.96 - 0.80 (m, 74 H).

^{13}C-NMR (125.8 MHz, $CDCl_3$, r.t.) δ [ppm]: 155.13, 154.46, 154.30, 153.85, 153.71, 153.70, 153.67, 153.65, 153.51, 153.48, 153.46, 153.43, 153.39, 153.24, 151.46, 150.54, 132.26, 128.75, 119.92, 118.11, 117.98, 117.96, 117.93, 117.41, 117.39, 117.37, 117.32, 117.24, 117.21, 117.19, 116.82, 116.66, 116.57, 115.65, 115.54, 115.10, 114.95, 114.77, 114.52, 114.49, 114.39, 114.34, 114.27, 114.13, 113.79, 113.43, 112.75, 112.74, 112.64, 92.56, 92.49, 91.79, 91.70, 91.55, 91.44, 70.00, 69.99, 69.91, 69.89, 69.85, 69.83, 69.81, 69.80, 69.77, 69.75, 69.73, 69.52, 69.29, 69.21, 32.08, 31.92, 31.92, 31.88, 31.80, 31.79, 31.78, 31.76, 31.75, 31.74, 31.73, 31.69, 31.68, 31.58, 29.85, 29.56, 29.54, 29.51, 29.50, 29.48, 29.46, 29.44, 29.40, 29.37, 29.36, 29.34, 29.30, 29.28, 29.28, 29.24, 26.06, 26.01, 25.94, 25.86, 25.85, 25.83, 25.82, 25.80, 25.79, 25.78, 25.76, 25.70, 22.85, 22.84, 22.83, 22.80, 22.78, 22.75, 22.74, 22.73, 21.46, 20.92, 18.39, 18.15, 14.27, 14.23, 14.20, 14.19, 14.17, 14.16, 11.97, 9.80.

MS (MALDI-pos, DCTB) m/z = 4034.9 $[M]^+$, 4284.4 $[M + DCTB]^+$, 4535.0 $[M + 2 DCTB]^+$.

10a

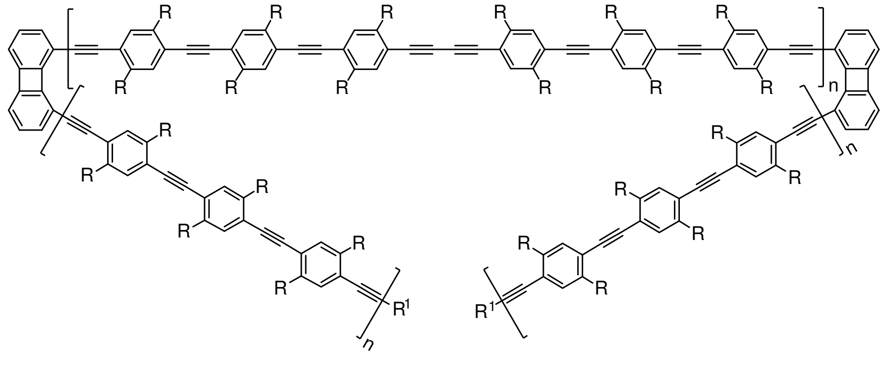

10a (n = 1) R^1 = CPDIPS, R = OC_6H_{13}

9a (38 mg, 17 µmol), $PdCl_2(PPh_3)_2$ (2 mg, 3 µmol), CuI (2 mg, 10 µmol) und iodine (3 mg, 12 µmol) were dissolved in THF (4 mL) and diisopropylamine (2 mL) and stirred at r.t. for 24 h. The reaction mixture was diluted with dichloromethane and water and the aqueous phase was

extracted with dichloromethane. The combined organic phase was washed with hydrochloric acid (2 M), water and brine and dried over magnesium sulfate. After removal of the solvent, the crude product was purified by column chromatography (cyclohexane:dichloromethane 1:1, R_f = 0.18 (2:3)) to give **10a** (34 mg, 8 µmol, 90 %) as a yellow film.

Formula: $C_{292}H_{388}N_2O_{24}Si_2$, molecular weight: 4366.5 g/mol.

^{1}H-NMR (500 MHz, $CDCl_3$, r.t.) δ [ppm]: 6.99 - 6.95 (m, 12 H), 6.94 (s, 2 H), 6.93 - 6.88 (m, 10 H), 6.77 (dd, J = 6.8 Hz, J = 1.7 Hz, 4 H), 6.76 (s, 4 H), 6.60 (d, J = 6.8 Hz, 4 H), 4.04 - 3.92 (m, 32 H), 3.89 (t, J = 6.4 Hz, 8 H), 3.47 (t, J = 6.5 Hz, 8 H), 2.43 (t, J =7.0 Hz, 4 H), 1.93 - 1.74 (m, 44 H), 1.70 - 1.63 (m, 8 H), 1.55 - 1.43 (m, 44 H), 1.42 - 1.24 (m, 112 H), 1.15 - 1.09 (m, 28 H), 0.97 - 0.76 (m, 64 H).

^{13}C-NMR (125.8 MHz, $CDCl_3$, r.t.) δ [ppm]: 155.11, 154.46, 153.84, 153.70, 153.65, 153.64, 153.50, 153.45, 153.36, 151.46, 150.54, 132.25, 128.74, 119.91, 117.95, 117.92, 117.30, 117.21, 116.82, 116.65, 116.54, 115.64, 115.04, 114.76, 114.64, 114.53, 114.52, 114.27, 114.21, 114.12, 113.78, 113.76, 113.33, 112.64, 104.13, 95.21, 92.52, 92.09, 92.03, 91.86, 91.79, 91.53, 91.44, 90.06, 79.48, 69.97, 69.87, 69.82, 69.75, 69.74, 69.71, 69.51, 69.26, 69.21, 32.07, 31.92, 31.87, 31.78, 31.75, 31.74, 31.69, 29.84, 29.53, 29.50, 29.47, 29.45, 29.44, 29.35, 29.27, 26.00, 25.94, 25.84, 25.82, 25.80, 25.78, 25.77, 22.82, 22.79, 22.77, 22.74, 21.45, 20.91, 18.39, 18.15, 14.26, 14.22, 14.20, 14.17, 11.96, 9.79.

MS (MALDI-pos, DCTB) m/z = 4366.3 $[M]^+$, 4616.6 $[M + DCTB]^+$.

10b

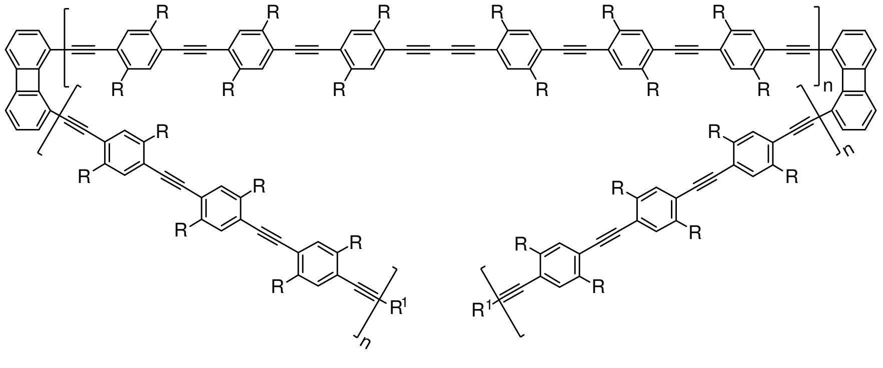

10b (n = 2) R^1 = CPDIPS, R = OC_6H_{13}

9b (27 mg, 6.6 µmol), $PdCl_2(PPh_3)_2$ (0.37 mg, 0.5 µmol), CuI (0.13 mg, 0.7 µmol) und iodine (0.84 mg, 3.3 µmol) were dissolved in THF (4 mL) and diisopropylamine (4 mL) and stirred at r.t. for 16 h. The reaction mixture was diluted with dichloromethane and water and the aqueous phase was extracted with dichloromethane. The combined organic phase was washed with hydrochloric acid (2 M), water and brine and dried over magnesium sulfate. After removal of the

solvent, the crude product was purified by recGPC and subsequently by column chromatography (cyclohexane:dichloromethane 2:3, R_f = 0.16) to give **10b** (18 mg, 2.2 µmol, 68 %) as a yellow film.

Formula: $C_{540}H_{724}N_2O_{48}Si_2$, molecular weight: 8067.9 g/mol.

^{1}H-NMR (500 MHz, $CDCl_3$, r.t.) δ [ppm]: 7.03 - 6.94 (m, 40 H), 6.94 - 6.88 (m, 8 H), 6.80 - 6.74 (m, 8 H), 6.60 (d, J = 6.8 Hz, 4 H), 4.09 - 3.93 (m, 72 H), 3.90 (t, J = 6.5 Hz, 16H), 3.47 (t, J = 6.5 Hz, 8 H), 2.43 (t, J = 7.0 Hz, 4 H), 1.93 - 1.73 (m, 100 H), 1.70 - 1.62 (m, 8 H), 1.60 - 1.44 (m, 88 H), 1.44 - 1.21 (m, 192 H), 1.19 - 1.06 (m, 28 H), 0.96 - 0.76 (m, 148 H).

^{13}C-NMR (125.8 MHz, $CDCl_3$, r.t.) δ [ppm]: 155.14, 154.47, 153.86, 153.71, 153.66, 153.51, 153.49, 153.47, 153.40, 151.47, 150.55, 132.27, 128.76, 119.92, 117.99, 117.95, 117.41, 117.33, 117.25, 117.22, 116.84, 116.68, 116.59, 115.67, 115.56, 114.96, 114.78, 114.53, 114.47, 114.36, 114.28, 114.14, 113.80, 113.44, 112.76, 112.65, 104.11, 95.28, 92.50, 92.34, 92.10, 91.79, 91.69, 91.55, 91.44, 90.08, 79.79, 79.53, 79.47, 70.01, 69.92, 69.90, 69.86, 69.84, 69.83, 69.77, 69.75, 69.53, 69.31, 69.22, 31.93, 31.88, 31.79, 31.77, 31.76, 31.74, 31.70, 29.85, 29.81, 29.54, 29.52, 29.49, 29.46, 29.38, 29.36, 29.29, 26.01, 25.95, 25.85, 25.83, 25.81, 25.78, 22.83, 22.80, 22.79, 22.76, 21.46, 20.92, 18.40, 18.16, 14.27, 14.23, 14.21, 14.18, 14.13, 11.97, 9.81, 1.17.

MS (MALDI-pos, DCTB) m/z = 8073.4 $[M]^+$, 8323.1 $[M + DCTB]^+$, 8574.0 $[M + 2\ DCTB]^+$, 8824.7 $[M + 3\ DCTB]^+$, 9073.6 $[M + 4\ DCTB]^+$, 9323.5 $[M + 5\ DCTB]^+$.

11a

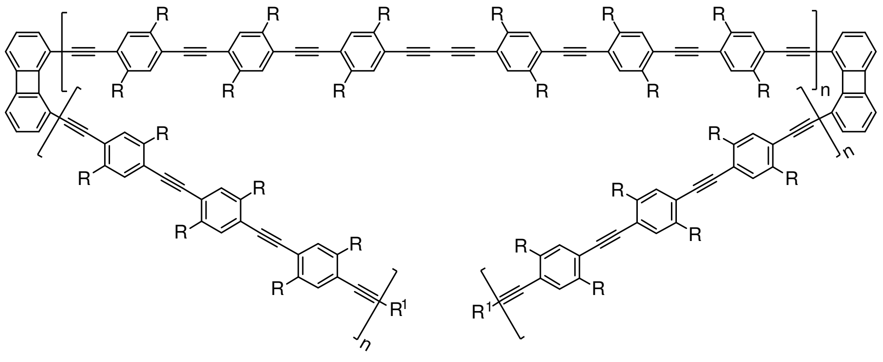

11a (n = 1) R^1 = H, R = OC_6H_{13}

10a (34 mg, 8 µmol) was dissolved in THF (6 mL) and a 1 M solution of TBAF in THF (0.02 mL, 20 µmol) was added. The reaction was stirred at room temperature for 90 min before it was diluted with water and dichloromethane. The aqueous phase was extracted with dichloromethane and the combined organic phase was washed with water and brine and dried over magnesium sulfate. After removal of the solvent, the crude product was purified by flash

chromatography (cyclohexane:dichloromethane 1:1, R_f = 0.30 (2:3)) to give **11a** (27 mg, 6.7 µmol, 87 %) as a yellow-orange film.

Formula: $C_{272}H_{35}O_{24}$, molecular weight: 4003.8 g/mol.

^{1}H-NMR (500 MHz, $CDCl_3$, r.t.) δ [ppm]: 7.00 - 6.94 (m, 16 H), 6.93 - 6.88 (m, 8 H), 6.80 - 6.74 (m, 8 H), 6.60 (d, J =6.8 Hz, 4 H), 4.04 - 3.93 (m, 32 H), 3.89 (t, J = 6.5 Hz, 8 H), 3.47 (t, J =6.5 Hz, 8 H), 3.34 (s, 2 H), 1.87 - 1.76 (m, 40 H), 1.71 - 1.62 (m, 8-H), 1.55 - 1.44 (m, 44-H), 1.42 - 1.22 (m, 112 H), 0.94 - 0.79 (m, 60 H).

^{13}C-NMR (125.8 MHz, $CDCl_3$, r.t.) δ [ppm]: 155.12, 154.30, 153.84, 153.71, 153.68, 153.64, 153.50, 153.45, 153.44, 151.47, 150.55, 132.26, 128.75, 118.08, 117.95, 117.31, 117.23, 117.20, 117.17, 116.83, 116.64, 115.64, 115.19, 114.77, 114.69, 114.53, 114.52, 114.28, 114.18, 114.13, 113.78, 113.77, 112.65, 92.50, 92.09, 92.03, 91.84, 91.79, 91.45, 91.30, 90.05, 82.39, 80.22, 79.76, 79.49, 69.88, 69.86, 69.82, 69.75, 69.72, 69.51, 69.21, 32.08, 31.93, 31.88, 31.83, 31.79, 31.74, 31.69, 29.85, 29.81, 29.72, 29.54, 29.51, 29.48, 29.46, 29.42, 29.39, 29.36, 29.33, 29.30, 29.28, 26.01, 25.96, 25.85, 25.83, 25.79, 25.78, 25.76, 22.84, 22.83, 22.80, 22.78, 22.75, 22.74, 22.68, 14.27, 14.23, 14.21, 14.19, 14.17, 14.16.

MS (MALDI-pos, DCTB) m/z = 4003.8 [M]$^+$, 4252.2 [M + DCTB]$^+$.

11b

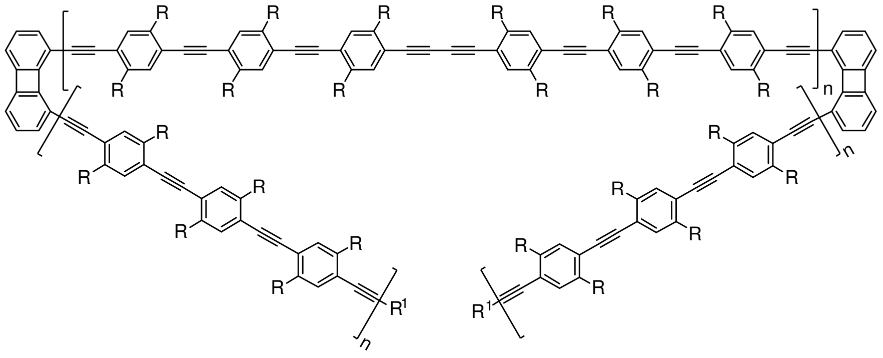

11b (n = 2) R^1 = H, R = OC_6H_{13}

10b (18 mg, 2.2 µmol) was dissolved in THF (6 mL) and a 1 M solution of TBAF in THF (0.06 mL, 20 µmol) was added. The reaction was stirred at room temperature for 16 h before it was diluted with water and dichloromethane. The aqueous phase was extracted with dichloromethane and the combined organic phase was washed with water and brine and dried over magnesium sulfate. After removal of the solvent, the crude product was purified by flash chromatography (cyclohexane:dichloromethane 2:3, R_f = 0.33) to give **11b** (14 mg, 1.8 µmol, 81 %) as an orange film.

Formula: $C_{520}H_{686}O_{48}$, molecular weight: 7705.2 g/mol.

^{1}H-NMR (500 MHz, $CDCl_3$, r.t.) δ [ppm]: 7.02 - 6.95 (m, 40 H), 6.92 (s, 4 H), 6.90 (d, J =8.2 Hz, 4 H), 6.79 - 6.75 (m, 8-H), 6.60 (d, J = 6.8 Hz, 4 H), 4.00 (m, 80 H), 3.90 (t, J = 6.5 Hz, 8H), 3.47 (t, J =6.5 Hz, 8 H), 3.34 (s, 2 H), 1.88 - 1.77 (m, 96 H), 1.55 - 1.47 (m, 96 H), 1.40 - 1.29 (m, 192 H), 0.96 - 0.76 (m, 144-H).

^{13}C-NMR (125.8 MHz, $CDCl_3$, r.t.) δ [ppm]: 155.13, 154.31, 153.86, 153.73, 153.72, 153.70, 153.68, 153.65, 153.51, 153.49, 153.46, 151.47, 150.55, 132.27, 128.76, 118.12, 117.99, 117.97, 117.42, 117.33, 117.25, 117.21, 116.83, 116.67, 115.66, 115.54, 115.11, 114.77, 114.53, 114.50, 114.39, 114.28, 114.14, 113.79, 112.74, 112.64, 69.92, 69.90, 69.86, 69.84, 69.78, 69.76, 69.74, 69.69, 69.52, 69.21, 32.09, 31.99, 31.93, 31.88, 31.83, 31.81, 31.80, 31.79, 31.77, 31.76, 31.75, 31.70, 31.69, 29.90, 29.86, 29.81, 29.59, 29.55, 29.52, 29.50, 29.49, 29.47, 29.45, 29.43, 29.40, 29.38, 29.36, 29.35, 29.30, 29.28, 29.25, 29.15, 26.03, 26.02, 25.88, 25.88, 25.85, 25.83, 25.81, 25.79, 25.78, 25.76, 22.88, 22.85, 22.84, 22.81, 22.79, 22.79, 22.76, 22.74, 22.72, 14.28, 14.28, 14.23, 14.21, 14.19, 14.18, 14.17, 14.16, 1.17.

MS (MALDI-pos, DCTB) m/z = 7705.0 $[M]^+$, 7956.1 $[M + DCTB]^+$, 8203.5 $[M + 2\ DCTB]^+$.

2

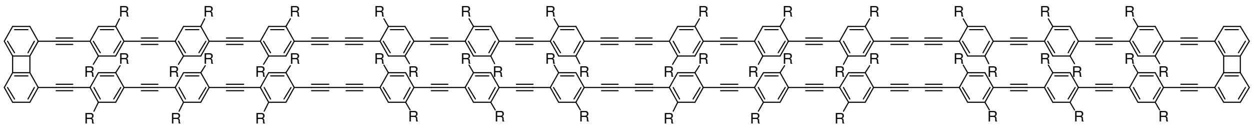

2, R = OC_6H_{13}

$PdCl_2(PPh_3)_2$ (10 mg, 14 µmol), CuI (1.7 mg, 9 µmol) und iodine (2.3 mg, 9 µmol) were dissolved in THF (10 mL) and diisopropylamine (10 mL) and heated to 40 °C. A solution of **11b** (13.7 mg, 1.8 µmol) in THF (15 mL) was slowly added to the catalyst system over 24 h and stirred for a further 16 h at this temperature. The reaction mixture was diluted with dichloromethane and water and the aqueous phase was extracted with dichloromethane. The combined organic phase was washed with hydrochloric acid (2 M), water and brine and dried over magnesium sulfate. After removal of the solvent, the crude product was purified by filtration through a plug of silica and subsequently by recGPC to give **2** (4.5 mg, 0.6 µmol, 33 %) as a yellow-orange film.

Formula: $C_{540}H_{684}O_{48}$, molecular weight: 7703.1 g/mol.

^{1}H-NMR (700 MHz, $CDCl_3$, r.t.) δ [ppm]: 7.02 - 6.94 (m, 40 H), 6.92 - 6.89 (m, 8 H), 6.77 (t, J = 7.5 Hz, 4 H), 6.74 (s, 4 H), 6.60 (d, J = 7.0 Hz, 4 H), 4.05 - 3.96 (m, 72 H), 3.94 (t, J = 6.6 Hz, 8 H), 3.87 (t, J = 6.4 Hz, 8 H), 3.47 (t, J = 7.5 Hz, 8 H), 1.87 - 1.77 (m, 96 H), 1.57 - 1.45 (m, 96 H), 1.40 - 1.28 (m, 192 H), 0.95 - 0.80 (m, 144 H).

^{13}C-NMR (176 MHz, $CDCl_3$, r.t.) δ [ppm]: 155.16, 155.13, 153.84, 153.76, 153.71, 153.62, 153.53, 153.49, 153.45, 151.47, 150.55, 117.99, 117.93, 117.41, 117.24, 69.92, 69.89, 69.86, 69.83, 69.77, 69.71, 69.49, 69.46, 69.21, 45.35, 32.78, 32.09, 31.95, 31.93, 31.89, 31.82, 31.80, 31.77, 31.76, 31.74, 31.70, 31.23, 29.87, 29.60, 29.54, 29.50, 29.48, 29.47, 29.45, 29.44, 29.40, 29.38, 29.36, 29.30, 29.28, 29.18, 26.73, 26.01, 25.86, 25.86, 25.84, 25.83, 25.81, 25.78, 22.89, 22.85, 22.83, 22.82, 22.79, 22.76, 22.74, 22.73, 22.65, 14.28, 14.28, 14.24, 14.23, 14.20, 14.18, 14.17, 14.13, 14.09.
MS (MALDI-pos, DCTB) *m/z* = 7704.6 $[M]^+$, 7954.1 $[M + DCTB]^+$, 8202.8 $[M + 2\ DCTB]^+$, 8454.3 $[M + 3\ DCTB]^+$, 8704.6 $[M + 4\ DCTB]^+$.

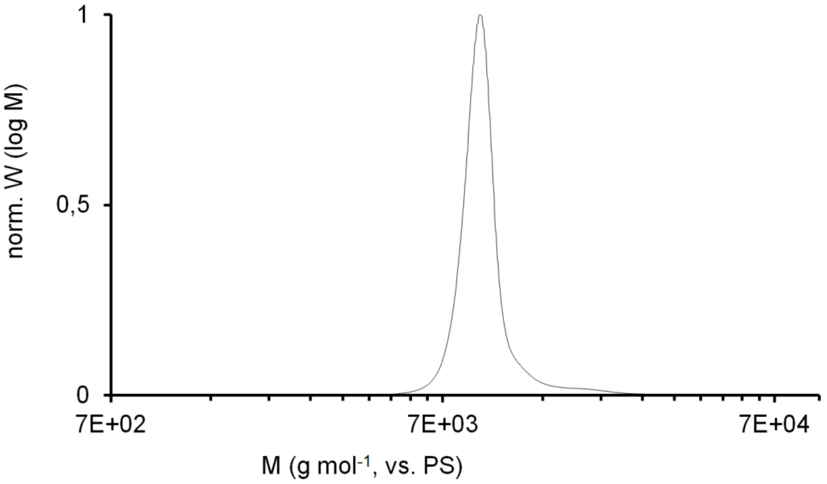

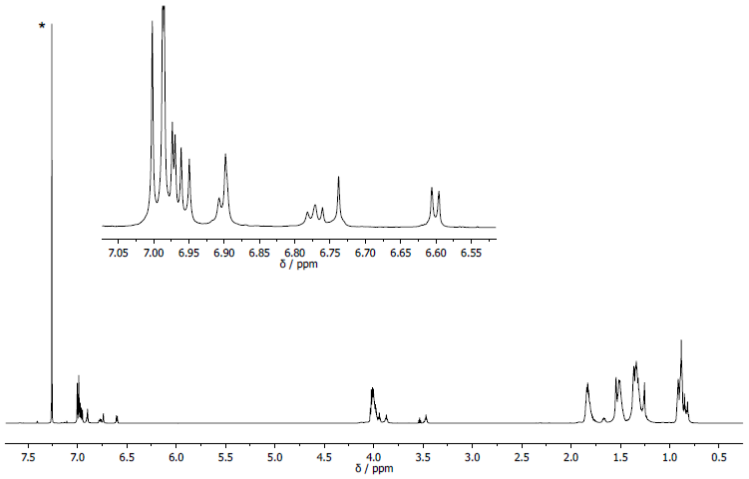

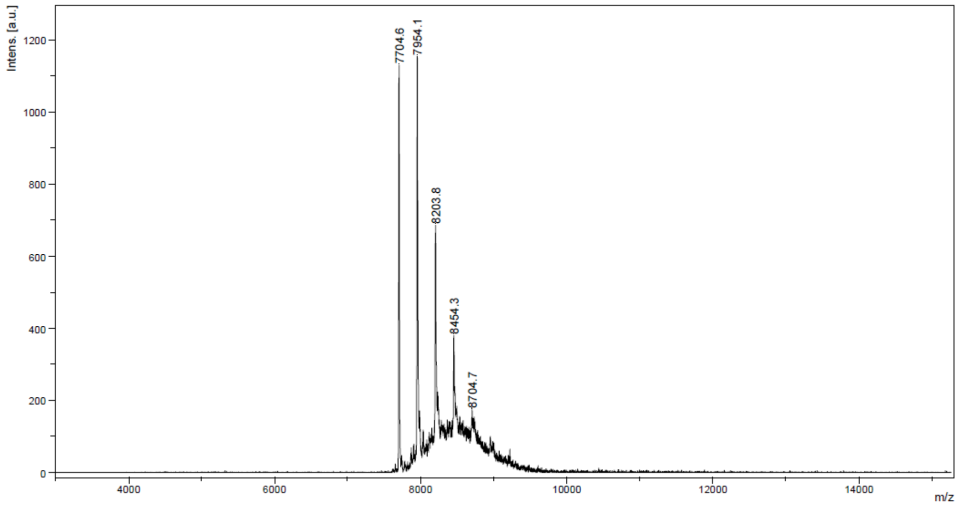

Figure S3: Analytical GPC, ^{1}H-NMR ($CDCl_3$) spectrum and MALDI spectrum of **2** (from top to down).

3

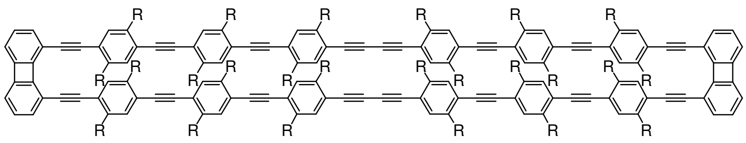

3, R = OC_6H_{13}

$PdCl_2(PPh_3)_2$ (47 mg, 67 µmol), CuI (6.4 mg, 33 µmol) und iodine (20 mg, 78 µmol) were dissolved in THF (20 mL) and diisopropylamine (30 mL) and heated to 40 °C. A solution of **11b** (27 mg, 6.7 µmol) in THF (40 mL) was slowly added to the catalyst system over 60 h and stirred for a further 11 h at r.t. The reaction mixture was diluted with dichloromethane and water and the aqueous phase was extracted with dichloromethane. The combined organic phase was washed with hydrochloric acid (2 M), water and brine and dried over magnesium sulfate. After removal of the solvent, the crude product was purified by filtration through a plug of silica and subsequently by recGPC to give **3** (6 mg, 1.5 µmol, 22 %) as a yellow-orange film.

Formula: $C_{272}H_{348}O_{24}$, molecular weight: 4001.8 g/mol.

^{1}H-NMR (400 MHz, $CDCl_3$, r.t.) δ [ppm]: 6.93 (s, 4 H), 6.92 (s, 4 H), 6.91 (s, 4 H), 6.90 (d, J =8.4 Hz, 4 H), 6.89 (s, 4 H), 6.84 (s, 4 H), 6.77 (dd, J = 8.4 Hz, J = 6.8 Hz, 4 H), 6.69 (s, 4 H), 6.60 (d, J = 6.8 Hz, 4 H), 4.03 - 3.88 (m, 32 H), 3.85 (t, J = 6.6 Hz, 4 H), 3.80 (t, J = 6.5 Hz, 4 H), 3.50 (t, J = 6.7 Hz, 8 H), 1.85 - 1.72 (m, 40 H), 1.73 - 1.64 (m, 8 H), 1.53 - 1.41 (m, 44 H), 1.42 - 1.22 (m, 112 H), 0.94 - 0.79 (m, 60 H).

^{13}C-NMR (125.8 MHz, $CDCl_3$, r.t.) δ [ppm]: 155.12, 153.75, 153.54, 153.49, 153.40, 151.63, 150.57, 132.23, 128.73, 117.82, 117.14, 116.89, 116.32, 115.63, 114.77, 114.55, 114.32, 113.61, 112.68, 92.04, 91.60, 69.84, 69.79, 69.76, 69.59, 69.41, 69.21, 31.99, 31.90, 31.88, 31.78, 31.74, 29.86, 29.53, 29.51, 29.38, 29.32, 26.01, 25.87, 25.83, 25.81, 25.79, 22.84, 22.83, 22.81, 22.80, 22.78, 14.28, 14.26, 14.22, 14.21.

MS (MALDI-pos, DCTB) m/z = 4001.8 $[M]^+$, 4252.4 $[M + DCTB]^+$, 4503.1 $[M + 2\ DCTB]^+$, 4753.2 $[M + 3\ DCTB]^+$.

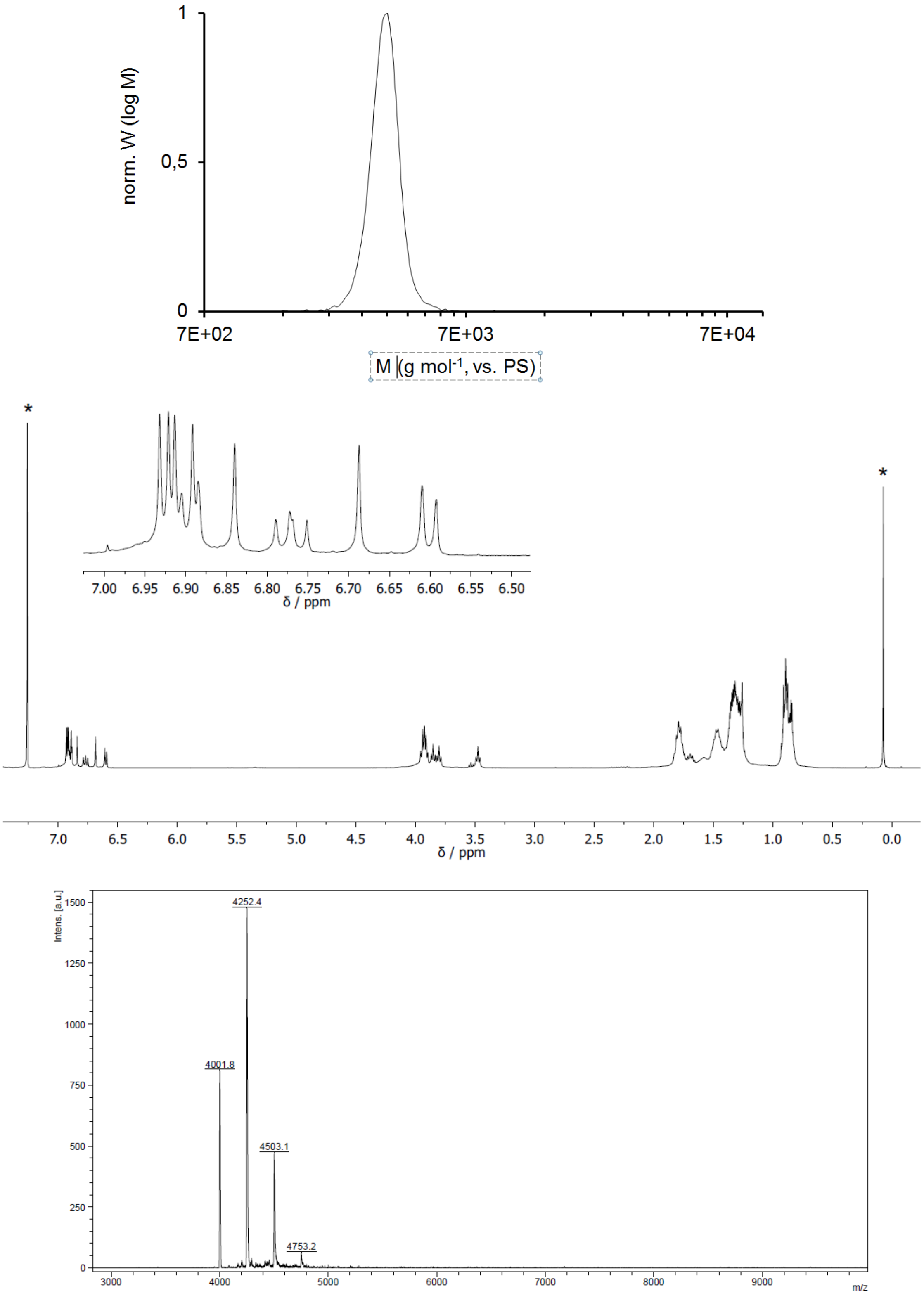

Figure S4: Analytical GPC, ^{1}H-NMR ($CDCl_3$) spectrum and MALDI spectrum of **3** (from top to down).

1.5 Additional compounds

Figure S5 gives an overview of the additional bichromophoric compounds **12** and **13** and the trichromophoric compound **14**.

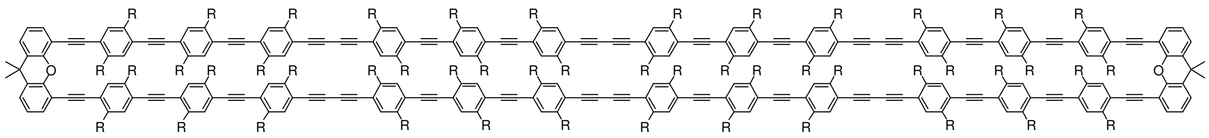

12, R = OC_6H_{13}

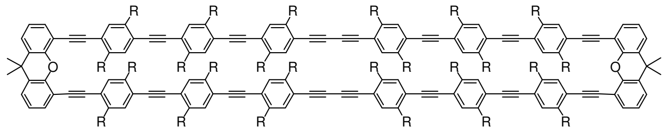

13, R = OC_6H_{13}

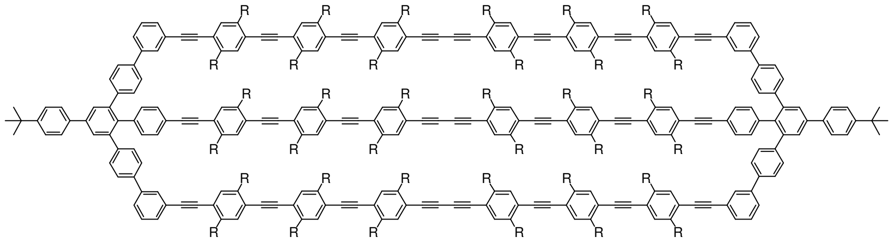

14, R = OC_6H_{13}

Figure S5. Structures of **12, 13** and **14.**

1.5 Synthesis

The synthesis **13** and **14** has been described before[4,5]. The syntheses of **12** is performed according to the synthesis of **2** and **3**.

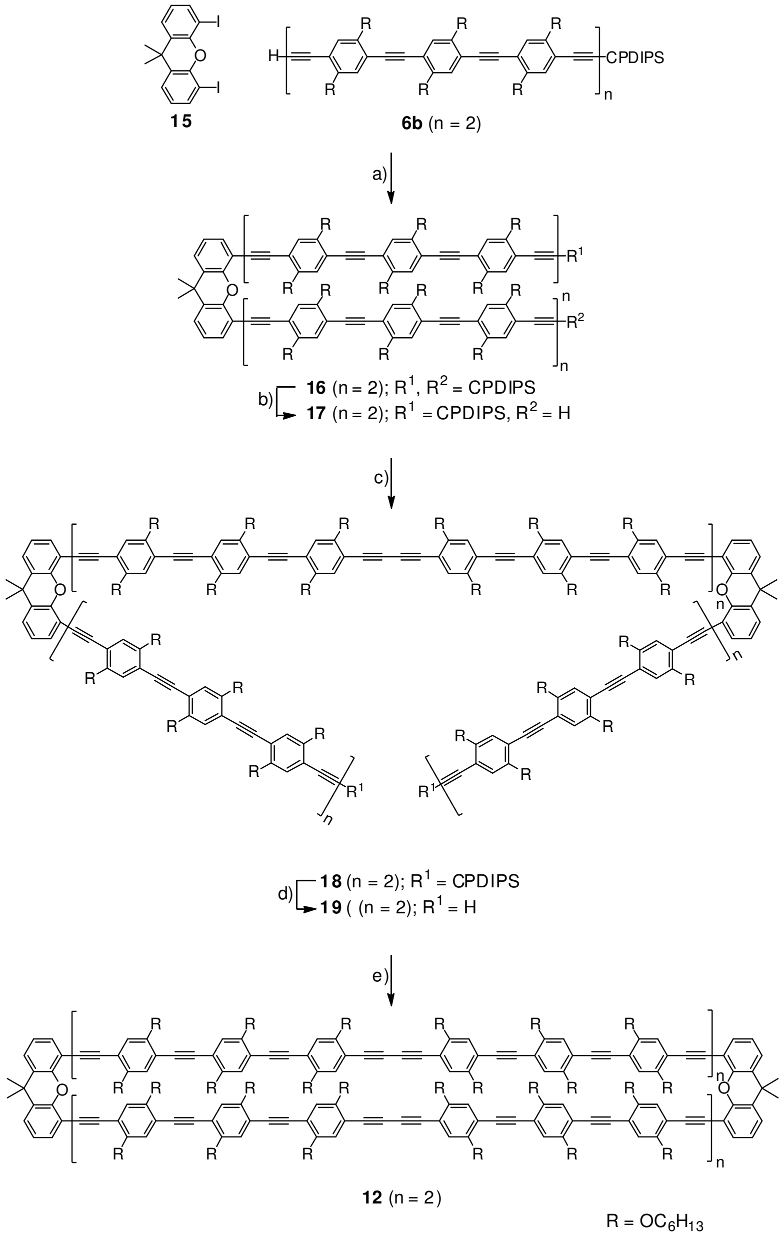

Scheme S1c. a) $PdCl_2(PPh_3)_2$, CuI, PPh_3, piperidine, THF, microwave, 120 °C, 20 min, 71 %; b) TBAF, THF, water, r.t., 3:05 h, 42 %; c) $PdCl_2(PPh_3)_2$, CuI, I_2, THF, diisopropylamine, r.t., 16 h, 90 %; d) TBAF, THF, r.t., 6 h, 75 %; e) $PdCl_2(PPh_3)_2$, CuI, I_2, THF, diisopropylamine, 40 °C, 40 h, 48 %.

16

Under an argon atmosphere, **15**[6] (9.7 mg, 21 µmol) and **6b**[1] (89.7 mg, 44 µmol), $PdCl_2(PPh_3)_2$ (1 mg, 1 µmol), CuI (1 mg, 5 µmol) and PPh_3 (1 mg, 4 µmol) were placed in a microwave tube and THF (5 mL) and piperidine (2 mL) were added and the mixture was heated to 120 °C for 20

min and then stirred at r.t. over night. Contrary to the literature, **15** was purified by column chromatography and subsequent HPLC to be obtained as a colorless crystalline solid. The reaction was diluted with water and dichloromethane. The aqueous phase was extracted with dichloromethane and the combined organic phase was washed with hydrochloric acid (2 M), water and brine and dried over magnesium sulfate. After removal of the solvent, the crude product was purified by column chromatography (cyclohexane:dichloromethane 1:1, R_f = 0.15 (2:5)) and subsequently by rec GPC to give **16** (53.9 mg, 15 µmol, 71 %) as an orange film.

Formula: $C_{283}H_{388}N_2O_{25}Si_2$, molecular weight: 4273.4 g/mol.

^{1}H-NMR (500 MHz, $CDCl_3$, r.t.) δ [ppm]: 7.49 (dd, *J* =7.7 Hz, *J* =1.6 Hz, 2H), 7.42 (dd, *J* =7.7 Hz, *J* =1.6 Hz, 2H), 7.11 (t, *J* =7.7 Hz, 2H), 7.05 - 6.93 (m, 24H), 4.15 - 3.91 (m, 44H), 3.57 (t, *J* =6.5 Hz, 4H), 2.46 (t, *J* =7.0 Hz, 4H), 1.99 - 1.76 (m, 52H), 1.68 (s, 6H), 1.61 - 1.46 (m, 48H), 1.44 - 1.23 (m, 96H), 1.19 - 1.05 (m, 28H), 0.97 - 0.79 (m, 76H).

^{13}C-NMR (125.6 MHz, $CDCl_3$, r.t.) δ [ppm]: 155.13, 155.12, 154.46, 153.73, 153.70, 153.66, 153.61, 153.48, 153.45, 153.39, 150.19, 132.20, 130.24, 123.05, 119.90, 117.97, 117.94, 117.38, 117.24, 117.20, 116.93, 116.58, 115.72, 115.54, 114.97, 114.96, 114.52, 114.37, 114.36, 114.17, 113.98, 113.43, 112.77, 112.62, 112.59, 104.10, 95.27, 92.57, 92.32, 91.72, 91.55, 91.51, 91.36, 91.24, 79.81, 79.54, 79.44, 69.99, 69.91, 69.88, 69.84, 69.81, 69.69, 69.62, 69.29, 69.23, 37.24, 34.35, 32.90, 32.59, 32.07, 31.94, 31.89, 31.81, 31.78, 31.76, 31.74, 31.70, 31.69, 31.58, 30.34, 30.18, 29.84, 29.80, 29.63, 29.53, 29.50, 29.48, 29.46, 29.45, 29.44, 29.37, 29.29, 29.28, 27.23, 26.01, 25.94, 25.86, 25.84, 25.82, 25.80, 25.79, 25.78, 22.84, 22.80, 22.78, 22.75, 21.46, 20.91, 19.88, 18.39, 18.15, 14.26, 14.23, 14.21, 14.19, 14.18, 11.96, 9.80, 1.16.

MS (MALDI-pos, DCTB) m/z = 4274.4 $[M]^+$, 4524.6 $[M+DCTB]^+$, 4776.3 $[M+2DCTB]^+$.

17

Under an argon atmosphere, **16** (63.9 mg, 15 µmol) was dissolved in THF (3.0 mL) and water (0.15 mL). A 1 M solution of TBAF in THF (0.04 mL, 40 µmol) was added. The reaction was stirred and monitored by TLC. After 30 min, additional TBAF in THF (0.04 mL, 40 µmol) was added, 30 min later again (0.04 mL, 40 µmol), 45 min later again (0.04 mL, 40 µmol), and 35 min later again (0.02 mL, 20 µmol). After a total reaction time of 3:05 h the mixture was diluted with water and dichloromethane. The aqueous phase was extracted with dichloromethane and the combined organic phase was washed with water and brine and dried over magnesium sulfate. After removal of the solvent, the crude product was purified by column chromatography

(cyclohexane:dichloromethane 1:1 -> 1:3, R_f = 0.28 (1:3)) to give **17** (25.7 mg, 6 µmol, 42 %) as a yellow solid.

Formula: $C_{273}H_{369}NO_{25}Si$, molecular weight: 4093.0 g/mol.

^{1}H-NMR (700 MHz, $CDCl_3$, r.t.) δ [ppm]: 7.49 (dd, J =7.7 Hz, J =1.5 Hz, 2H), 7.42 (dd, J =7.7 Hz, J =1.6 Hz, 2H), 7.11 (t, J =7.7 Hz, 2H), 7.05 - 6.92 (m, 24H), 4.10 - 3.93 (m, 44H), 3.56 (t, J =6.6 Hz, 4H), 3.36 (s, 1H), 2.46 (t, J =7.0 Hz, 2H), 1.92 - 1.77 (m, 50H), 1.68 (s, 6H), 1.61 - 1.48 (m, 48H), 1.43 - 1.26 (m, 96H), 1.18 - 1.10 (m, 14H), 0.97 - 0.81 (m, 74H).

^{13}C-NMR (176 MHz, $CDCl_3$, r.t.) δ [ppm]: 155.13, 155.12, 154.47, 154.30, 153.73, 153.70, 153.67, 153.66, 153.65, 153.64, 153.61, 153.48, 153.47, 153.45, 153.39, 150.19, 138.00, 132.21, 130.25, 129.17, 128.36, 126.33, 125.43, 123.05, 119.92, 118.12, 118.11, 118.10, 117.98, 117.96, 117.93, 117.42, 117.39, 117.37, 117.24, 117.22, 117.21, 117.19, 116.92, 116.86, 116.58, 115.72, 115.53, 115.11, 114.97, 114.95, 114.52, 114.49, 114.39, 114.37, 114.35, 114.17, 113.98, 113.43, 112.77, 112.74, 112.63, 112.58, 92.58, 92.33, 92.30, 91.74, 91.71, 91.56, 91.52, 91.36, 91.24, 70.00, 69.91, 69.88, 69.84, 69.83, 69.82, 69.80, 69.79, 69.77, 69.75, 69.73, 69.69, 69.67, 69.62, 69.57, 69.29, 69.23, 68.11, 34.36, 32.60, 32.08, 31.97, 31.94, 31.91, 31.89, 31.86, 31.84, 31.81, 31.80, 31.79, 31.77, 31.76, 31.75, 31.74, 31.73, 31.70, 31.69, 31.66, 29.89, 29.85, 29.81, 29.69, 29.65, 29.63, 29.59, 29.57, 29.54, 29.50, 29.48, 29.46, 29.45, 29.44, 29.40, 29.37, 29.36, 29.35, 29.29, 29.28, 29.26, 29.22, 27.09, 27.07, 27.06, 26.08, 26.03, 26.01, 25.94, 25.90, 25.87, 25.86, 25.84, 25.83, 25.82, 25.80, 25.79, 25.78, 25.76, 25.74, 25.68, 22.90, 22.88, 22.85, 22.81, 22.81, 22.78, 22.75, 22.74, 22.72, 22.67, 22.66, 22.64, 21.46, 20.92, 18.39, 18.15, 14.27, 14.27, 14.24, 14.22, 14.20, 14.19, 14.18, 14.16, 14.14, 11.97, 9.80, 1.17, 1.16.

MS (MALDI-pos, DCTB) m/z = 4092.9 $[M]^+$, 4343.0 $[M + DCTB]^+$.

18

17 (25.7 mg, 6 µmol), $PdCl_2(PPh_3)_2$ (0.35 mg, 0.5 µmol), CuI (0.12 mg, 0.6 µmol) und iodine (0.80 mg, 0.6 µmol) were dissolved in THF (4 mL) and diisopropylamine (4 mL) and stirred at r.t. for 16 h. The reaction mixture was diluted with dichloromethane and water and the aqueous phase was extracted with dichloromethane. The combined organic phase was washed with hydrochloric acid (2 M), water and brine and dried over magnesium sulfate. After removal of the solvent, the crude product was purified by rec GPC and then by column chromatography (cyclohexane:dichloromethane 2:5, R_f = 0.62) to give **18** (17.9 mg, 2 µmol, 90 %) as an orange film.

Formula: $C_{546}H_{736}N_2O_{50}Si_2$, molecular weight: 8184.0 g/mol.

^{1}H-NMR (700 MHz, $CDCl_3$, r.t.) δ [ppm]: 7.47 (dd, J =7.6 Hz, J =1.6 Hz, 4H), 7.40 (dd, J =7.6 Hz, J =1.6 Hz, 4H), 7.08 (t, J =7.6 Hz, 4H), 7.03 - 6.90 (m, 48H), 4.09- 3.89 (m, 88H), 3.54 (t, J =6.3 Hz, 8H), 2.43 (t, J = 7.0 Hz, 4H), 1.95 - 1.75 (m, 100H), 1.67 (s, 12H), 1.59 - 1.44 (m, 96H), 1.42 - 1.23 (m, 192H), 1.16 - 1.06 (m, 28H), 0.96 - 0.81 (m, 148H).

^{13}C-NMR (176 MHz, $CDCl_3$, r.t.) δ [ppm]: 155.14, 155.12, 154.47, 153.73, 153.70, 153.67, 153.65, 153.63, 153.61, 153.49, 153.45, 153.40, 153.40, 150.19, 132.21, 130.25, 123.05, 119.92, 118.17, 117.98, 117.97, 117.93, 117.40, 117.37, 117.25, 117.22, 117.20, 117.16, 116.92, 116.86, 116.58, 115.72, 115.53, 114.97, 114.95, 114.52, 114.38, 114.35, 114.16, 113.97, 113.43, 112.77, 112.63, 112.59, 70.00, 69.92, 69.89, 69.86, 69.85, 69.83, 69.81, 69.69, 69.62, 69.30, 69.23, 34.36, 32.60, 32.08, 31.95, 31.94, 31.90, 31.84, 31.82, 31.81, 31.79, 31.77, 31.76, 31.75, 31.74, 31.70, 31.65, 29.85, 29.66, 29.63, 29.60, 29.56, 29.54, 29.51, 29.50, 29.48, 29.46, 29.45, 29.44, 29.37, 29.36, 29.30, 29.28, 29.26, 29.21, 26.05, 26.05, 26.02, 25.95, 25.95, 25.87, 25.86, 25.85, 25.83, 25.82, 25.80, 25.78, 25.78, 25.65, 22.85, 22.85, 22.81, 22.79, 22.76, 22.75, 22.68, 22.65, 22.63, 21.46, 20.92, 18.40, 18.15, 14.27, 14.24, 14.22, 14.19, 14.18, 14.12, 11.97, 9.81, 1.18, 1.16.

MS (MALDI-pos, DCTB) m/z = 8184.0 $[M]^+$, 8435.6 $[M + DCTB]^+$, 8686.5 $[M+2DCTB]^+$.

19

18 (17.9 mg, 2.2 µmol) was dissolved in THF (4 mL) and a 1 M solution of TBAF in THF (5.5 µL, 5.5 µmol) was added. The reaction was stirred at room temperature for 6 h before it was diluted with water and dichloromethane. The aqueous phase was extracted with dichloromethane and the combined organic phase was washed with water and brine and dried over magnesium sulfate. After removal of the solvent, the crude product was purified by flash chromatography

(cyclohexane:dichloromethane 2:3, R_f = 0.41) to give **19** (12.8 mg, 1.7 µmol, 75 %) as an orange film.
Formula: $C_{526}H_{698}O_{50}$, molecular weight: 7821.3 g/mol.
^{1}H-NMR (700 MHz, $CDCl_3$, r.t.) δ [ppm]: 7.46 (dd, *J* =7.6 Hz, *J* =1.6 Hz, 4H), 7.40 (dd, *J* =7.6 Hz, *J* =1.6 Hz, 4H), 7.08 (t, *J* =7.6 Hz, 4H), 7.02 - 6.90 (m, 48H), 4.06 - 3.90 (m, 88H), 3.54 (t, *J* =6.7 Hz, 8H), 3.34 (s, 2H), 1.88 - 1.77 (m, 96H), 1.66 (s, 12H), 1.58 - 1.45 (m, 96H), 1.40 - 1.29 (m, 192H), 0.95 - 0.80 (m, 144H).
^{13}C-NMR (176.8 MHz, $CDCl_3$, r.t.) δ [ppm]: 155.13, 154.31, 153.86, 153.73, 153.72, 153.70, 153.68, 153.65, 153.51, 153.49, 153.46, 151.47, 150.55, 132.27, 128.76, 118.12, 117.99, 117.97, 117.42, 117.33, 117.25, 117.21, 116.83, 116.67, 115.66, 115.54, 115.11, 114.77, 114.53, 114.50, 114.39, 114.28, 114.14, 113.79, 112.74, 112.64, 69.92, 69.90, 69.86, 69.84, 69.78, 69.76, 69.74, 69.69, 69.52, 69.21, 32.09, 31.99, 31.93, 31.88, 31.83, 31.81, 31.80, 31.79, 31.77, 31.76, 31.75, 31.70, 31.69, 29.90, 29.86, 29.81, 29.59, 29.55, 29.52, 29.50, 29.49, 29.47, 29.45, 29.43, 29.40, 29.38, 29.36, 29.35, 29.30, 29.28, 29.25, 29.15, 26.03, 26.02, 25.88, 25.88, 25.85, 25.83, 25.81, 25.79, 25.78, 25.76, 22.88, 22.85, 22.84, 22.81, 22.79, 22.79, 22.76, 22.74, 22.72, 14.28, 14.28, 14.23, 14.21, 14.19, 14.18, 14.17, 14.16, 1.17.
MS (MALDI-pos, DCTB) *m/z* = 7705.0 $[M]^+$, 7956.1 $[M + DCTB]^+$, 8203.5 $[M + 2 DCTB]^+$.

12

$PdCl_2(PPh_3)_2$ (9.2 mg, 13 µmol), CuI (1.6 mg, 8 µmol) und iodine (2.1 mg, 8 µmol) were dissolved in THF (10 mL) and diisopropylamine (10 mL) and heated to 40 °C. A solution of **19** (12.8 mg, 1.6 µmol) in THF (15 mL) was slowly added to the catalyst system over 24 h and stirred for a further 16 h at r.t. The reaction mixture was diluted with dichloromethane and water and the aqueous phase was extracted with dichloromethane. The combined organic phase was washed with hydrochloric acid (2 M), water and brine and dried over magnesium sulfate. After removal of the solvent, the crude product was purified by filtration through a plug of silica and subsequently by recGPC to give **12** (6.1 mg, 0.8 µmol, 48 %) as a yellow film.
Formula: $C_{526}H_{696}O_{50}$, molecular weight: 7819.3 g/mol.
^{1}H-NMR (700 MHz, $CDCl_3$, r.t.) δ [ppm]: 7.47 (dd, *J* =7.8 Hz, *J* =1.7 Hz, 4H), 7.40 (dd, *J* =7.8 Hz, *J* =1.7 Hz, 4H), 7.08 (t, *J* =7.5 Hz 4H), 7.00 (s, 8H), 6.99 (s, 9H), 6.99 (s, 7H), 6.97 (s, 4H), 6.97 (s, 4H), 6.95 (s, 4H), 6.94 (s, 4H), 6.91 (s, 4H), 6.88 (s, 4H), 4.05 - 3.94 (m, 72H), 3.94 -- 3.89 (m, 16H), 3.56 - 3.50 (m, 8H), 1.92 - 1.74 (m, 96H), 1.71 - 1.63 (m, 12H), 1.59 - 1.43 (m, 96H), 1.43 - 1.19 (m, 192H), 0.97 - 0.79 (m, 144H).

^{13}C-NMR (176 MHz, $CDCl_3$, r.t.) δ [ppm]: 155.18, 155.14, 155.13, 153.77, 153.71, 153.68, 153.66, 153.60, 153.49, 153.47, 153.44, 150.21, 130.29, 123.07, 117.99, 117.93, 117.41, 117.35, 117.25, 117.21, 116.82, 112.58, 69.92, 69.83, 34.36, 32.59, 32.01, 31.97, 31.94, 31.91, 31.85, 31.84, 31.78, 31.76, 31.75, 31.72, 31.70, 29.63, 29.52, 29.50, 29.46, 29.40, 29.38, 29.36, 29.35, 29.31, 29.28, 26.06, 26.02, 25.88, 25.88, 25.86, 25.85, 25.84, 25.81, 25.80, 25.78, 22.85, 22.83, 22.81, 22.80, 22.76, 22.75, 22.65, 14.27, 14.26, 14.25, 14.20, 14.19, 14.15, 14.13, 1.18. MS (MALDI-pos, DCTB) *m/z* = 7819.3 $[M]^+$.

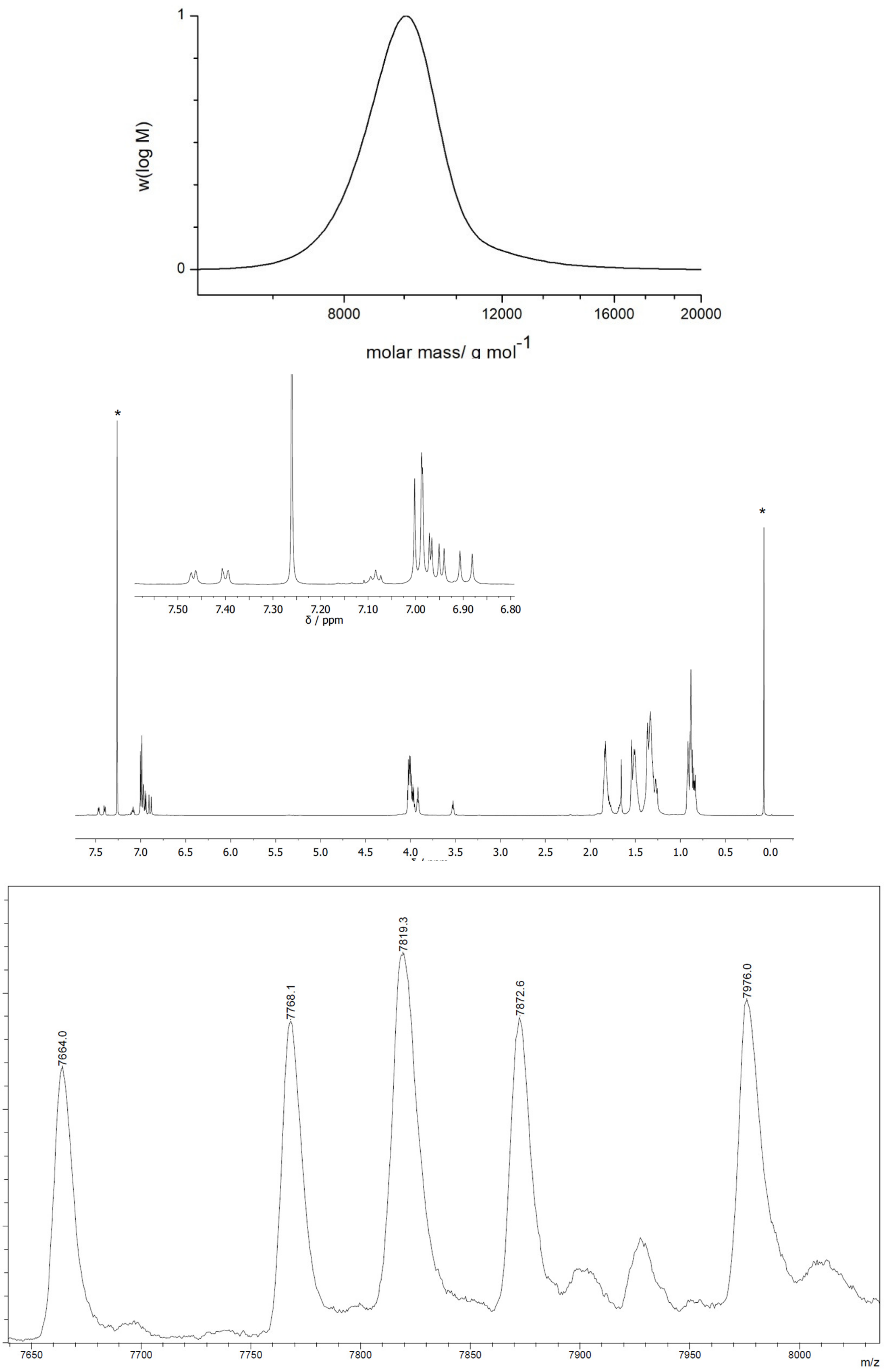

Figure S6: Analytical GPC, ^{1}H NMR (CD_2Cl_2) spectrum and MALDI spectrum of **12** (from top to down). The additional peaks in the MALDI-TOF spectrum arise from an internal PS calibration.

2. Scanning tunneling microscopy

Scanning tunneling microscopy (STM) images were acquired under ambient conditions (room temperature) at the solution/solid interface. A freshly cleaved piece of highly oriented pyrolytic graphite (HOPG) was heated to 80 °C on a hot plate. 0.5 µL of a 1×10^{-6} M (or 5×10^{-6} M) solution of the long dimer **2** or the monomer **1** in 1,2,4-trichlorobenzene (TCB) were dropped onto the hot HOPG surface, and it was kept at this temperature for 20 s before being allowed to cool to r.t. All STM measurements were performed at the solid/liquid-interface (i.e. with the tip immersed into the liquid) and typically completed within 30 minutes after sample preparation. A bias voltage of –0.8 V and a tunnelling current set point of 50 pA (monomer) or 40 pA (dimer) were applied to image the supramolecular adlayers shown here. The experimental setup consists of an Agilent 5500 AFM/STM, placed on a Halcyonics actively damping microscopy workstation, and is noise isolated with a home-built acoustic damping box. Mechanically cut Pt/Ir (80/20) tips were used and further modified *in situ* by applying short voltage pulses. HOPG was obtained from TipsNano via Anfatec, Germany in ZYB quality and freshly cleaved prior to each experiment. Unless otherwise specified, all STM images were calibrated *in situ* by subsequent immediate acquisition of an additional image at reduced bias voltage. Because of this process, the atomic lattice of the HOPG surface is visible and can be used as a calibration grid. Data processing, also for image calibration, was performed using the SPIP 5 (Image Metrology) software package.

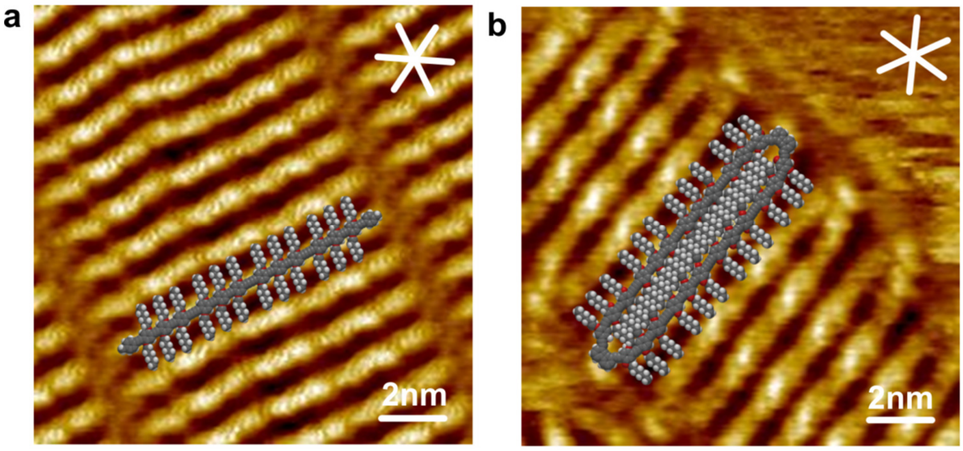

Figure S7. High-resolution scanning tunneling microscopy visualizes both the monomer **1** and the long dimer **2** at the solid/liquid interface of 1,2,4-trichlorobenzene and highly oriented pyrolytic graphite. The hexyloxy side chains of **1** interdigitate intermolecularly and are oriented along the substrate main axis directions so that the PEBs appear as bright stripes well-separated from each other. The p-tolyl end groups of adjacent molecules point towards each

other. Similarly, adjacent molecules of **2** interact *via* interdigitating side chains. However, 20 hexyloxy side chains interdigitate intraannularly and therefore deform the PEBs of the molecular backbone. The white asterisks indicate the HOPG main axis directions.

3. Ensemble measurements

To perform ensemble measurements in solution the analytes were dissolved in toluene and measured in 10 mm quartz cuvettes (Quartz SUPRASIL® from Hellma analytics). The absorption spectra were recorded using a Perkin Elmer spectrometer (Lambda 650). To obtain the emission spectra we used a Horiba Jobin-Yvon Fluoromax 4 fluorescence spectrometer at an excitation wavelength of 405 nm. The ensemble photoluminescence (PL) lifetime is extracted from streak camera (C5680-21 from Hamamatsu Photonics K.K.) measurements.

4. Single-molecule sample preparation and setup

To perform single-molecule measurements, the analyte molecules were embedded in a non-fluorescent polystyrene (PS, M_n=130 kDa, PDI=2.23 from Sigma Aldrich Co.) host matrix. To this end the following steps were carried out. First, borosilicate glass cover slips were sonicated (Elma, Elmasonic P) in a 2 % Hellmanex III (Hellma Analytics) solution, followed by rinsing with ultrapure MilliQ water. Second, the cover slips were transferred into a UV-ozone cleaner (Novoscan, PSD Pro Series UV) to bleach the glass coverslips from residual fluorescent molecules. Third, the analyte was diluted in toluene to single-molecule concentration (~10^{-12} M) and mixed with a 2 % PS/toluene solution. Fourth, this solution was then dynamically spin coated (Laurell Technologies Corporation, Model WS-400BZ-6NPP/LITE) at 2000 r.p.m. onto the glass cover slips, resulting in a film thickness of about 100 nm.

All measurements in the main text as well as for the supporting data were performed under ambient conditions. An inverted microscope (Olympus IX71) was used for confocal excitation of the samples and fluorescence detection. The excitation source was provided by a fiber-coupled diode laser (PicoQuant LDH-C-405) with a wavelength of 405 nm in pulsed mode with 80 MHz repetition rate. The laser light is passed through a clean-up filter (AHF analysentechnik AG, HC Laser Clean-up MaxDiode 405/10) and a λ/4 waveplate to obtain circularly polarized light. To perform the excitation polarization measurements in Figure S11, the linearly polarized excitation laser beam was rotated with an electro-optical modulator (FastPulse Technology Inc., 3079-

4PW) at a frequency of 40 Hz. The beam was expanded to a diameter of ~1 cm with a lens system and coupled into a high numerical aperture immersion objective (Olympus, UPLSAPO 60OX, NA = 1.35) by a dichroic mirror (AHF Analysentechnik AG, 405RDC). An area of 20×20 μm^2 is scanned using a piezo stage (Physik Instrumente (PI) GmbH & Co. KG) to obtain fluorescence images. An excitation intensity of ~200 W/cm^2 was used for photon antibunching and PL lifetime measurements and an intensity of ~400 W/cm^2 to obtain spectra. The fluorescence from the analyte molecules was collected by the objective and spatially and spectrally filtered by a 50 µm pinhole and fluorescence filter (AHF Analysentechnik AG, 405LP), to reduce background and laser influences. The filtered fluorescence was split with a 50:50 beam splitter into two equivalent detection channels. Two avalanche photodiodes from PicoQuant (τ-SPAD-20), connected to a time-correlated single-photon counting module (TCSPC, PicoQuant GmbH. HydraHarp 400) were used as detectors. A Hanbury-Brown and Twiss geometry is used to measure the time difference (Δt) between photon arrival times. From this data we also obtained PL decays. To extract the PL lifetime τ_{PL}, the decays were approximated by a single exponential decay function. Alternatively, we used a mirror to deflect the fluorescence signal into a spectrograph (Andor Technology plc., SR-303i-B) coupled with a CCD camera (Andor Technology plc., DU401A-BV) to obtain spectra from molecules which were subsequently placed inside the excitation focus. The PL lifetime, photon correlation and excitation polarization data were evaluated using a LabView program. The spectra were further analyzed by a customized software tool (Mathematica, Wolfram Research, Inc.) to extract the peak position, peak full width at half maximum and vibrational intensity ratio I_{0-0}/I_{0-1}.

5. Spectral fitting procedure

To analyze the measured spectra, we approximate them with multiple Gaussian functions. The first function approximates the 0-0 transition and therefore establishes the 0-0 peak energy. The next two Gaussians, which describe 0-1 bending and stretching modes of the C=C and C≡C bonds, have a fixed energy separation to the 0-0 peak as well as the same line width. To improve the fitting, the 0-2 transitions are also fitted by two further Gaussians at twice the energetic separation from the 0-0 peak. Finally, the sum of the two 0-1 vibrational peaks is also included in the fit, so that a total of 6 Gaussians are used to fit the spectrum. This approximation is shown in the figure below for two different example spectra. The three Gaussian peaks we ultimately use for further evaluation are marked with arrows. From this fitting procedure we gain the values of the 0-0 peak energy, the FWHM, $I_{0\text{-}0}$ as well as the Intensities of the two 0-1 vibrational modes which are used in the further evaluation. As seen in Fig. S8, the fit quality is quite good, even though it is not perfect. However, this automated procedure is perfectly sufficient to extract precise peak positions, intensities and FHWM values as used in the subsequent analysis. The automated procedure is used on thousands of measured spectra with no further manipulation of the fitting results.

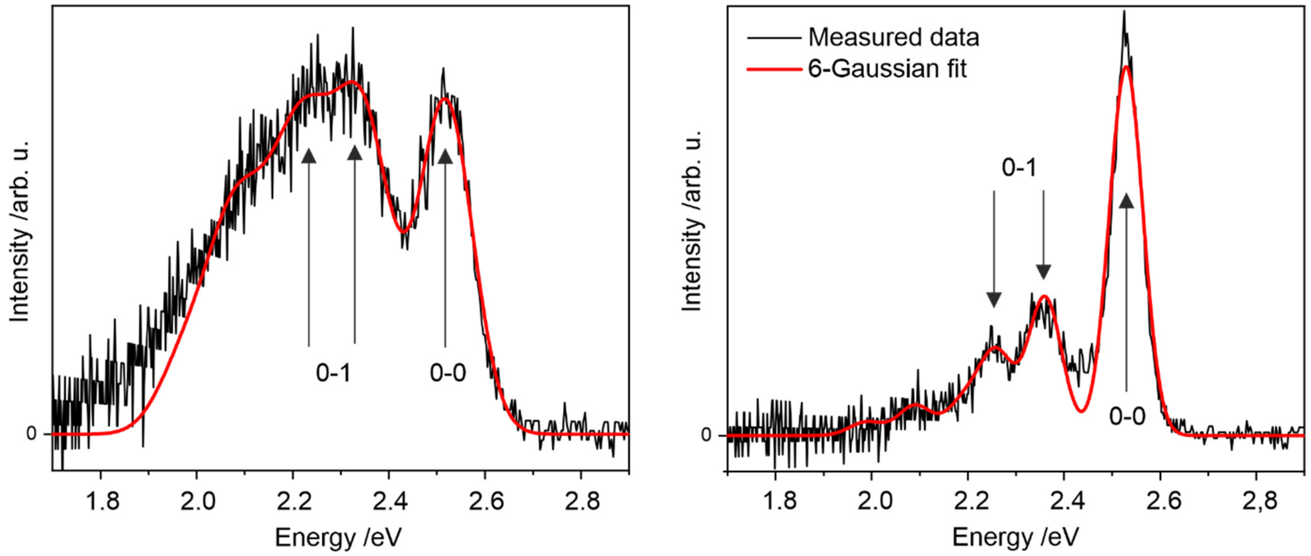

Figure S8. Two examples of automated peak fitting of the raw-data single-molecule spectra of the long dimer structure. A sum of a total of 6 Gaussians is used to fit the spectra, but only the 0-0 peak position and FWHM, and the 0-1 to 0-0 peak intensity ratio is used in the subsequent analysis.

6. Supporting figures

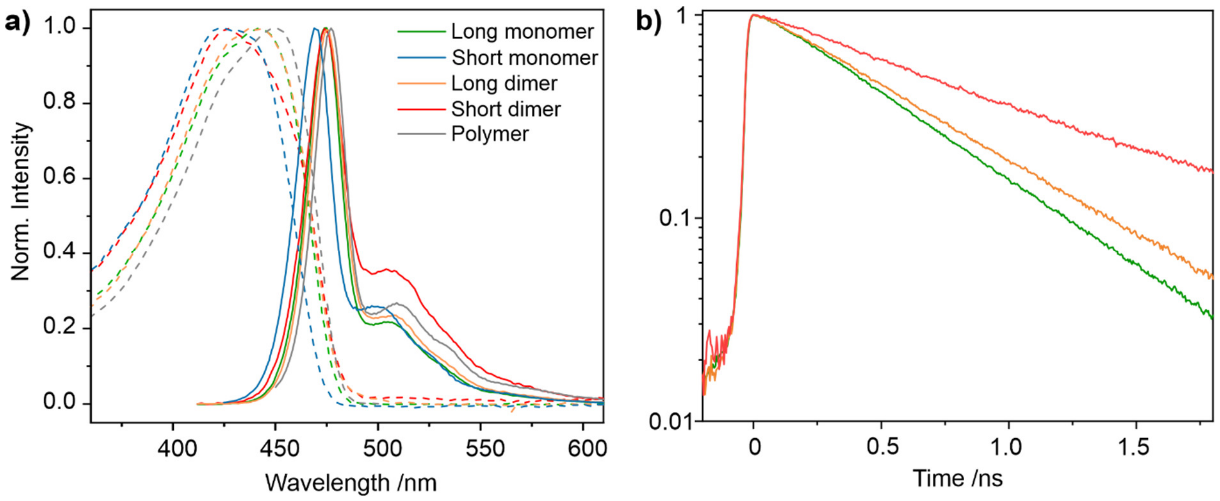

Figure S9. Ensemble measurements of the synthesized model systems in toluene solution. a) Absorption (dashed) and emission (solid) spectra of the three systems in addition to the 6-ring monomer and the ~240-ring polymer. The absorption of the 6-ring systems is slightly blue-shifted compared to the 12-ring systems due to the decreased delocalization of the π-system. In emission, the 12-ring systems show virtually identical spectra, whereas a small difference exists for the 6-ring systems. The red shift as well as the increase of the vibrational intensity of the 6-ring dimer compared to the 6-ring monomer can be assigned to the emergence of weak H-type coupling between the two cofacial chromophores of the dimer in solution. The end caps, which are only present in the dimers, appear to have no significant effect on the spectra, which can be seen by comparing the almost identical spectra of the 12-ring structures. The polymer sample shown contains approximately 40 repeat units of the small monomer and exhibits only a slight red shift of absorption and emission spectra compared to the 12-ring oligomer. This additional shift presumably arises because of a further increase in conjugation and therefore in the effective chromophore size. b) Streak-camera measured ensemble PL lifetime decays of the long monomer (green), long dimer (orange) and short dimer (red), respectively. By applying a single-exponential decay fit the PL lifetime can be extracted for the monomer as $\tau_{PL} = 0.55\ \mathrm{ns}$, for the long dimer $\tau_{PL} = 0.61\ \mathrm{ns}$ and the short dimer $\tau_{PL} = 0.84\ \mathrm{ns}$. The increase of the PL lifetime in the dimers can also be assigned to the presence of weak H-type coupling in the dimers. H-type coupling is expected to be stronger in the 6-ring dimer due to reduced flexibility of the segments.

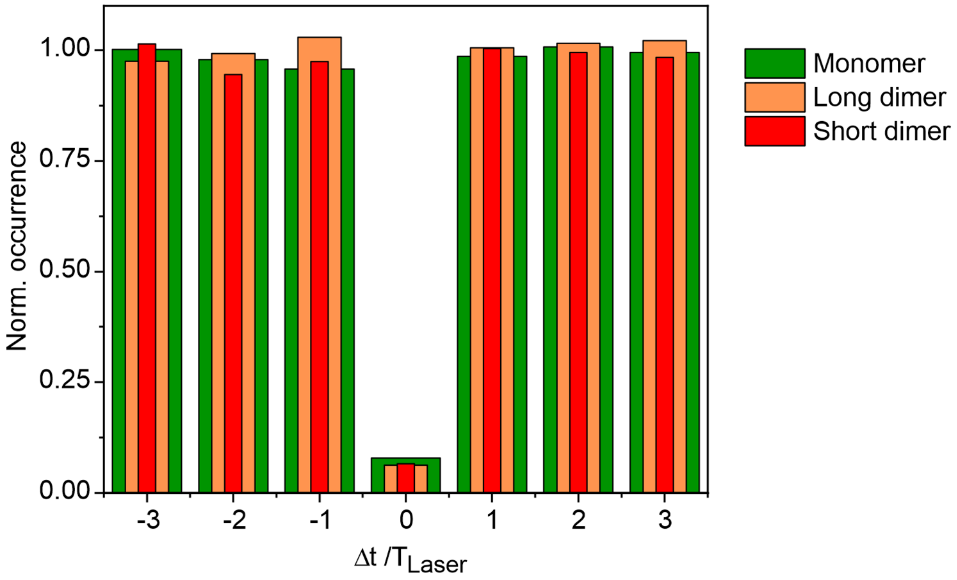

Figure S10. Photon antibunching histograms extracted from PL intensity traces of 62 single molecules of the 12-ring monomers (green), 109 12-ring dimers (orange) and 124 6-ring dimers (red) with the spacing of the bars being varied for clarity. The histogram measures the coincidence of photons passed through a beam splitter and detected by two photodiodes. The excitation laser was operated in a pulsed mode with a frequency of 80 MHz, which results in a time difference of 12.5 ns between two subsequent laser pulses (T_{Laser}). The histograms are normalized to the mean value of occurrences on the lateral peaks which describe photon arrival times with a time lag larger than one excitation pulse. The probability of measuring two photons at the detectors after the same laser pulse is then given by the central peak value which is 0.08 for the monomer, 0.06 for the 12-ring dimer and 0.07 for the 6-ring dimer. These values are close to the theoretical minimum for one emitter which is limited by the signal-to-noise ratio and therefore cannot be precisely zero.[7]

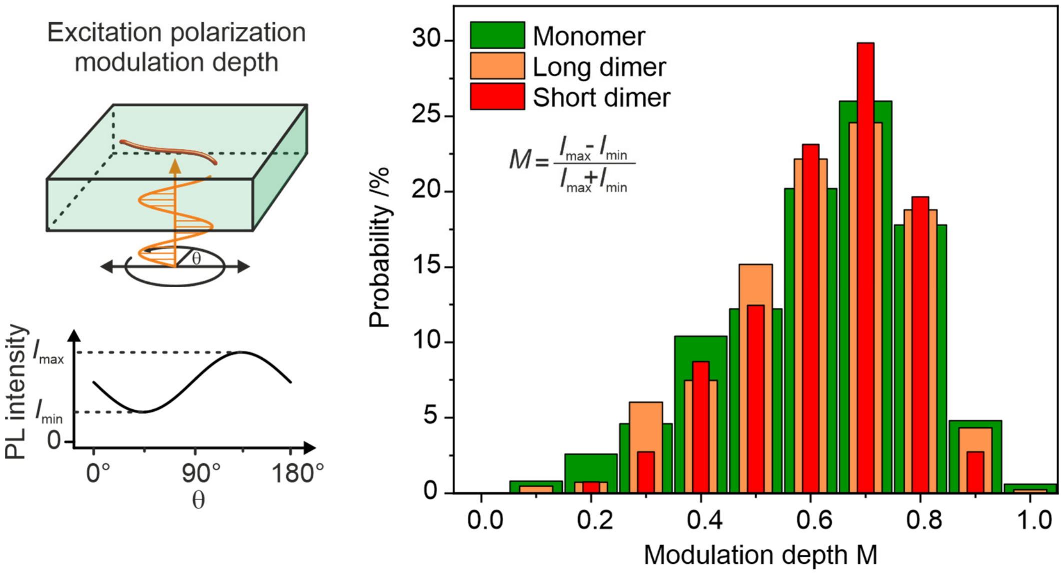

Figure S11. Examination of the polarization modulation depth in excitation of 500 single molecules of the 12-ring monomers, 414 molecules of the 12-ring dimers and 401 molecules of the 6-ring dimers. To examine the morphology, the excitation laser light is linearly polarized and rotated with an electro-optic modulator, which is schematically pictured on the left. For ordered molecules, the PL intensity will change with the rotation of the polarization of the excitation laser beam. From this intensity modulation, we extract the PL modulation depth (M). The histograms show a broad variation of M values for all three structures which means that the morphology ranges from bent to straight. No systematic difference is observed between the three distributions. As all distributions have a mean of M ≈ 0.62 we can deduce that most of the molecules are extended.

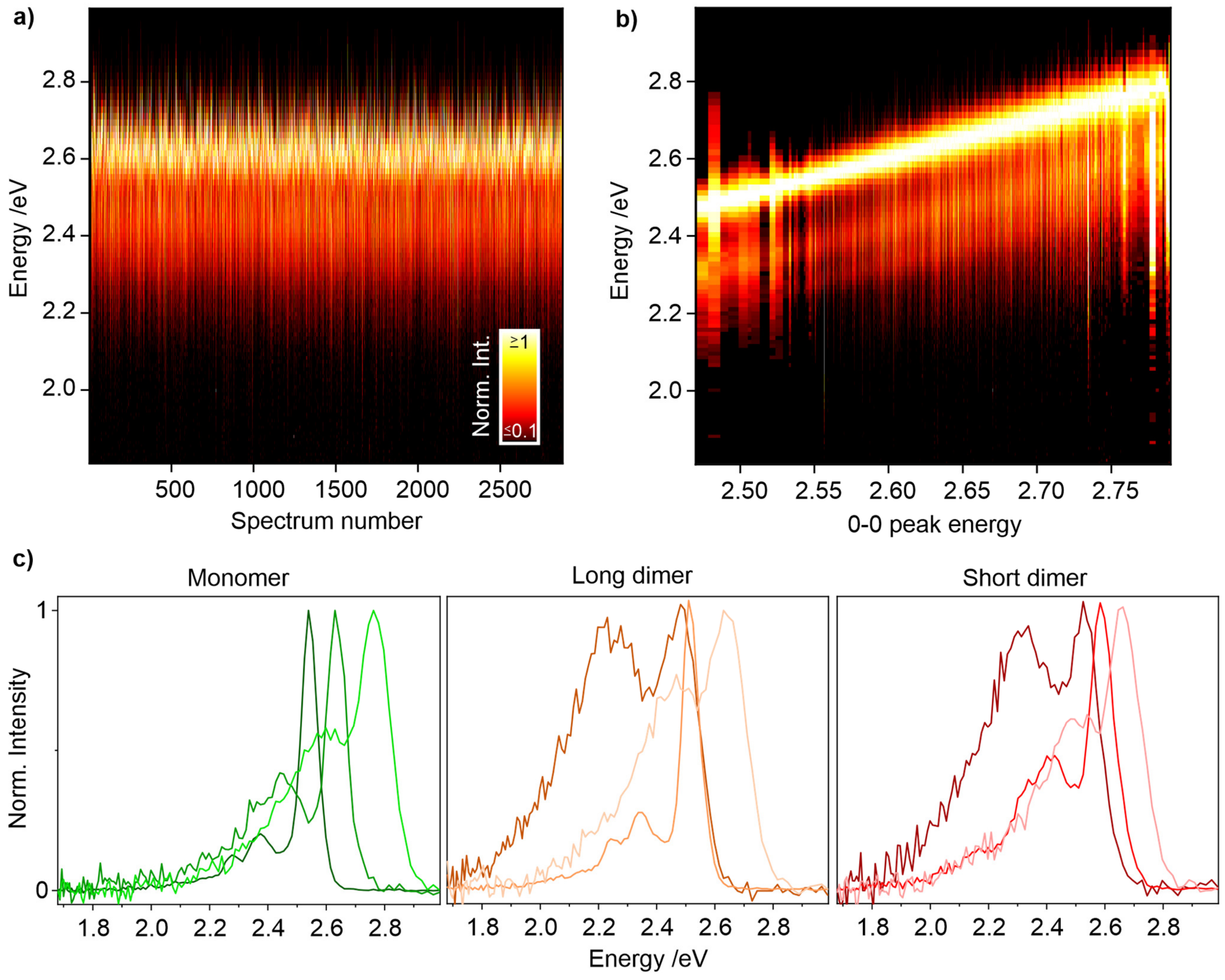

Figure S12. 2875 spectra of the 12-ring monomer before a) and after b) sorting by their 0-0 peak energy. All spectra are normalized to the intensity of the 0-0 peak. As described in Section 5, all measured spectra are approximated with a combination of Gaussians, which results in a value for the 0-0 peak energy of each spectrum. As H- or J-type coupling is always accompanied by a shift to lower emission energies, we sort the raw spectra a) by their 0-0 peak energies. With this method, plotted in b), we can uncover correlations in the raw data, which are not visible otherwise. In the unsorted case, all spectra appear to have the same width in the 2D plot as the points on the x-axis are equidistant. However, upon sorting the spectra by their 0-0 peak energy, this equal spacing between spectra disappears and some spectra appear broader depending how many spectra in the entire dataset exhibit the same peak energy. Examples of single-molecule PL spectra with different 0-0 peak energies are shown in c) for the 12-ring monomer as well as the 12 and 6-ring dimers. For all three samples, very similar spectral shapes are observed at a 0-0 peak transition energy of around 2.6 eV. These spectra have comparatively low 0-1 vibronic intensities and narrow peaks, and correspond to the most J-type transitions. For the monomer, these spectra are the red-most observed. Higher-energy transitions show stronger vibrational coupling and presumably arise from less well-ordered segments. Similar higher-energy spectra are also seen in the two dimer structures. However, in contrast to the monomer, the two dimers show clear signatures of H-aggregation in that the red-most spectra exhibit

a suppression of the 0-0 transition intensity as the relative 0-1 vibrational intensity is enhanced. The dimers therefore exhibit spectral signatures of both J-type intrachain and H-type interchain coupling.

Seeing how the influence of electronic coupling can indeed be discerned in the raw data, we constructed a sketch in Fig. 1b of the main text of what we expect for a model system exhibiting both H- and J-type coupling. For simplicity, we only considered the 0-0 and one vibronic transition, even though, of course, there are more characteristic vibrations in the molecule. The correlation is based on three effects: first, the spectral shift, characterized in our case by the 0-0 peak energy, should be stronger with increasing coupling strength. Second, the 0-1 to 0-0 peak intensity ratio increases with increasing H-type coupling strength but decreases with increasing J-type coupling strength. Third, the H-type coupling effects tend to dominate the J-type coupling effects if both effects occur together. We previously investigated this tradeoff in larger aggregates[4]. As the length of our model systems is well defined, there must be a maximum J-type coupling strength, i.e. a maximal possible red shift of the PL due to J-type aggregation. Further spectral red shifts must result from H-type coupling. At this point, the 0-1 to 0-0 PL peak ratio should increase suddenly as we enter the “H-type dominated“ regime marked in the sketch. Of course, there can also be H-type coupled molecules with lower 0-0 peak energies, which should appear in the right side of the sketch and which we refer to as “J-type” dominated. As we focus on emphasizing overall trends here, these spectra are not included in the sketch.

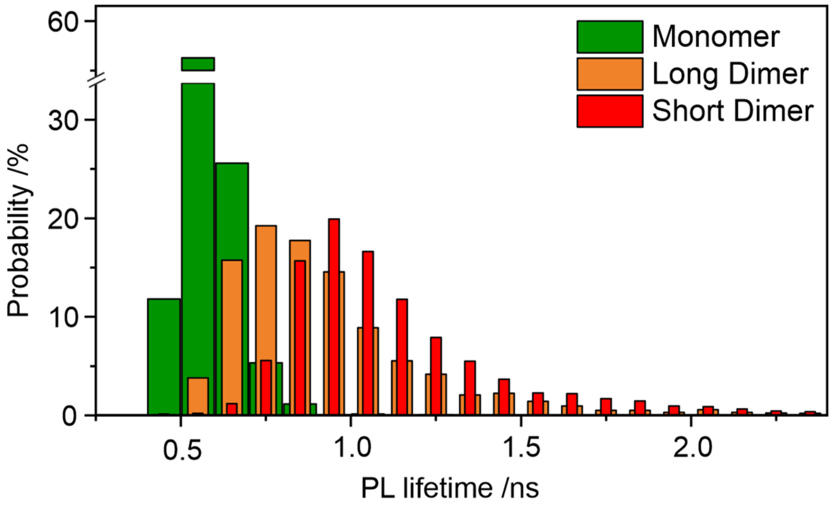

Figure S13. Histograms of the PL lifetime extracted from the PL decays of 1575 single molecules of the 12-ring monomers, 1606 molecules of the 12-ring dimers and 1367 molecules of the 6-ring dimers. The monomer lifetime (dark green) shows a narrow distribution with a mean value of $\tau_{PL} = 0.58\,\mathrm{ns}$, which is similar to the ensemble lifetime found in Figure S9. The PL lifetimes of the 12-ring dimer scatter more widely with a mean value of $\tau_{PL} = 0.95\,\mathrm{ns}$. This value is somewhat larger than the measured PL lifetime in solution, which we attribute to the increased strength of H-type coupling for molecules embedded in the matrix since this depends strongly on molecule conformation. For the short 6-ring dimers the PL lifetime histogram shifts further to higher values with a mean of $\tau_{PL} = 1.14\,\mathrm{ns}$, indicating even stronger coupling. In this case, the difference to the aforementioned PL lifetime in solution is also approx. 0.4 ns. Comparing the PL lifetimes of long and short dimers, it is evident how the length of the molecules impacts the interchromophoric coupling behavior. A large part of the long dimer population shows lifetimes lower than $0.75\,\mathrm{ns}$, which is not observed in the short dimers. The larger molecules are more strongly impacted by J-type coupling, as is seen by the fact that the lifetime distribution of the long dimers partially overlaps with that of the monomers. These differences between long and short dimers can be discerned even more clearly in the analysis of correlations between PL lifetime and 0-0 peak energy shown in Fig. S15.

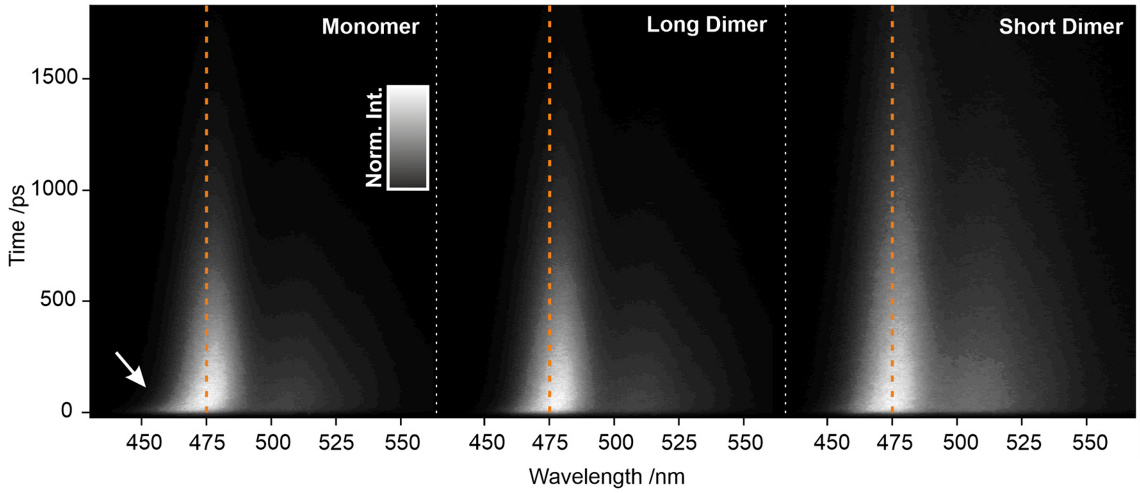

Figure S14. Streak camera images of the 12-ring monomer and the 12- and 6-ring dimers obtained in toluene solution. The normalized PL intensity is plotted versus wavelength and time. At early times a slight red shift can be seen in the monomer measurement (white arrow), which is weaker in the dimer measurements and can be attributed to structural relaxation in the excited state of the molecule. These images also show the increase of PL lifetime especially in the 6-ring dimer. In addition, the PL peak wavelengths obtained in the fluorescence spectrum of Figure S9a are also shown (orange). The extracted PL lifetime decays are plotted in Figure S9b.

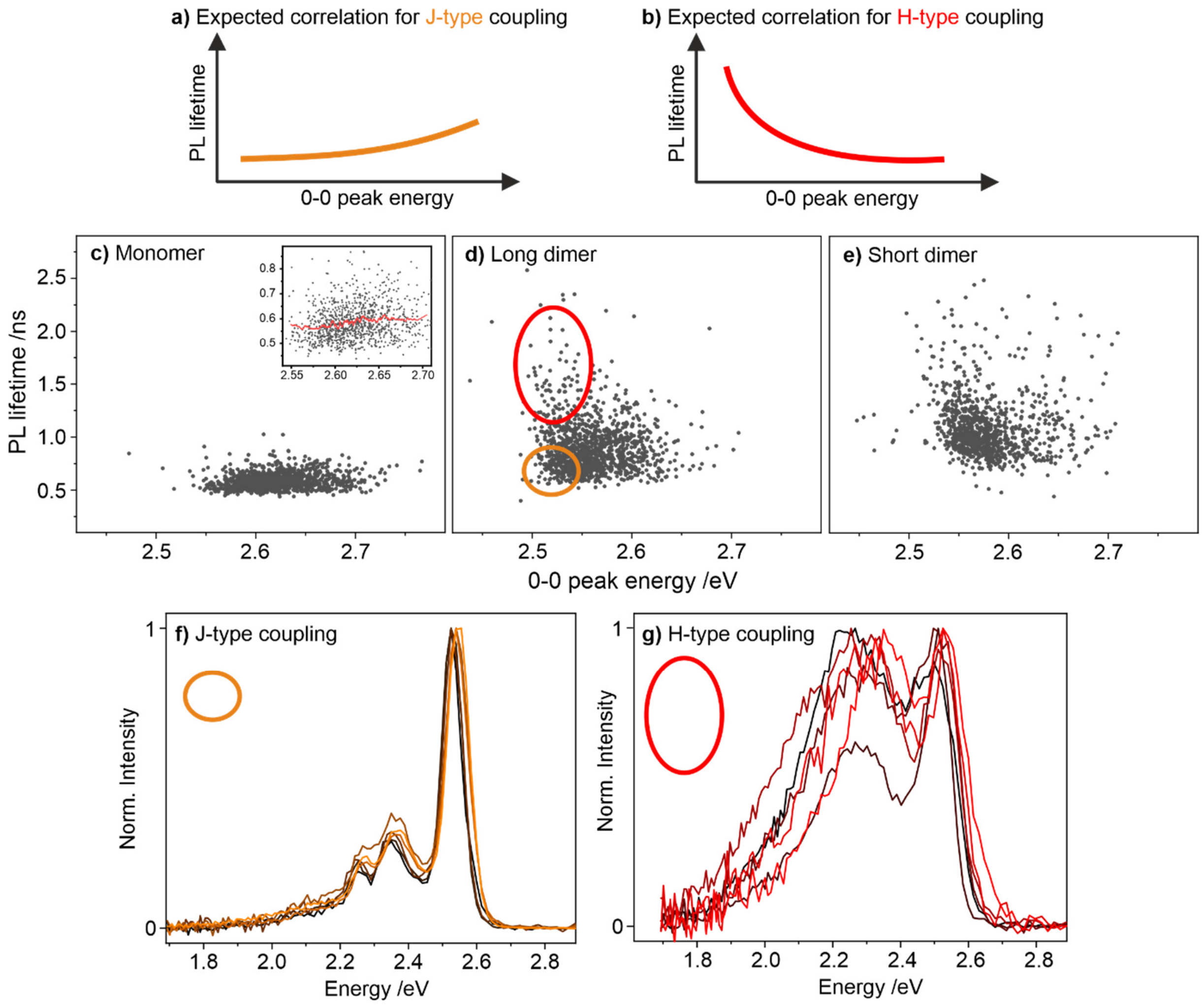

Figure S15. Correlations between the 0-0 peak energy and the PL lifetime. The upper part of the figure shows sketches of the anticipated correlation. The head-to-tail coupling of transition dipole moments leads to an increase in the oscillator strength as well as a spectral red shift[8]. J-type coupled molecules will exhibit a continuous decrease of the PL lifetime with decreasing 0-0 peak energy as the coupling strength increases, as indicated in panel a). The fewer monomers couple in a J-aggregate, the greater the change in radiative rate is expected to be, given the correlation a non-linear appearance. According to the theory of Kasha and Spano[8,9], H-type coupling leads to a spectral red shift in emission as well as the population of a dipole-forbidden state. This effect gives rise to the opposite correlation, an increase of the PL lifetime with spectral red shift. Since, in principle, a perfect H-aggregate can have an infinite radiative lifetime, orders of magnitude changes in lifetime can occur[10]. For the most strongly coupled aggregates, the correlation will therefore become very steep, as sketched in panel b)[11]. The greater the shift to lower 0-0 peak energies, the stronger the impact on the PL lifetime, which corresponds to increasing H-type coupling. The measured scatter plot of the 0-0 peak energy and PL lifetime of 1075 12-ring monomers in c) shows an overall small scatter in the PL lifetime. Crucially, however, a close inspection of the zoomed-in region of the scatter plot in the inset shows a slight decrease in PL lifetime with decreasing peak energy, a characteristic of the J-type coupling behavior in the monomer. The 994 6-ring dimers in e) show the expected behavior for H-type coupling. With decreasing 0-0 peak energy, the PL lifetime increases. The most interesting aspect is the scatter plot of 1192 measured 12-ring dimers in d), which shows a superposition of both H- and J-type correlations. As

elaborated on in the main text, because of the increased length compared to the 6-ring dimer, the 12-ring dimer can exhibit clear signatures of J-type coupling (i.e. equivalent to the monomer) if H-type coupling is not dominant. This population of strongly J-type coupled 12-ring dimers is marked with an orange circle in the lower left part of the lifetime-PL peak scatter plot. If the conformation of the dimer allows, H-type coupling arises and increases the PL lifetime again. In this case, the shift in 0-0 peak energy is similar to the shift in J-type coupled molecules. This population is marked in the plot by a red circle. Example spectra of both distinct regions of the correlation are given in panels f) and g): the J-type coupled molecules in f) show narrow spectra which are all very similar, whereas the H-type coupled molecules in g) show broad spectra which vary strongly in terms of the 0-1 to 0-0 PL peak intensity ratio.

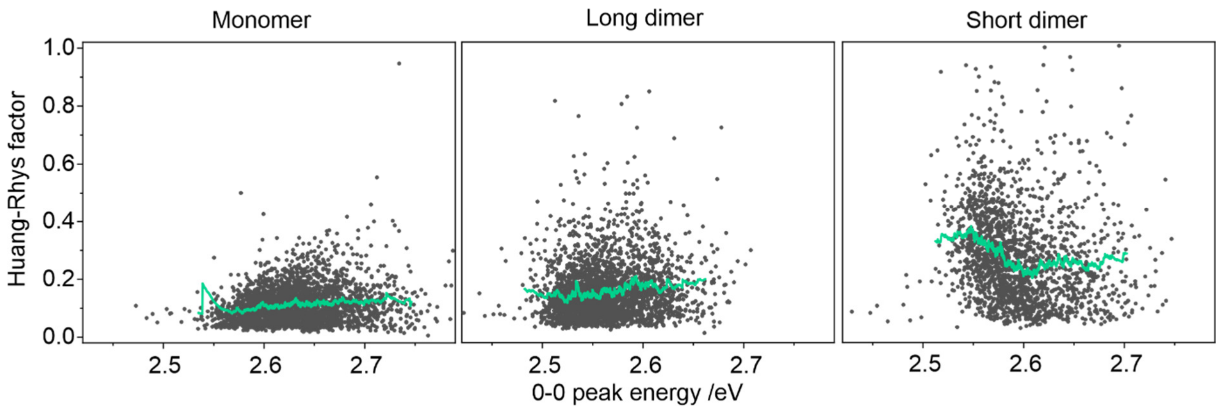

Figure S16. Huang-Rhys (HR) factors of the 0-1 vibrational modes of the three model systems. To visualize the trends, a 50-point moving average (green) is added in each scatter plot. A Franck-Condon analysis was performed to determine the HR factors. All spectra from Fig. 3 in the main text were approximated using Gaussian functions as described in Section 5 above, and the corresponding HR factors were extracted following the procedure described in Ref. [12]. Even though some spectra allow the differentiation between the different vibrational modes of the 0-1 vibrational feature, we show only the integrated HR factor of the overall 0-1 vibrations since the different vibrational modes merge with each other at room temperature. The 2875 molecules of the 12-ring monomer structure show a slight decrease of the HR factor with decreasing 0-0 peak energy, which is consistent with stronger J-type coupling for increasing conjugation length. This behavior does not change for the 2743 single molecules of the 12-ring dimers, where a slight overall decrease with decreasing 0-0 peak energy is also visible. In this case the scatter increases to higher HR values due to the influence of additional H-type coupling, which increases the 0-1 to 0-0 PL peak intensity ratio. As expected, the HR factors of the 1857 single molecules of the short 6-ring dimers are higher overall, which can be attributed to the shorter conjugation length. The increase for 0-0 peak energies lower than 2.6 eV arises due to H-type coupling, which increases the vibronic-to-electronic intensity ratio.

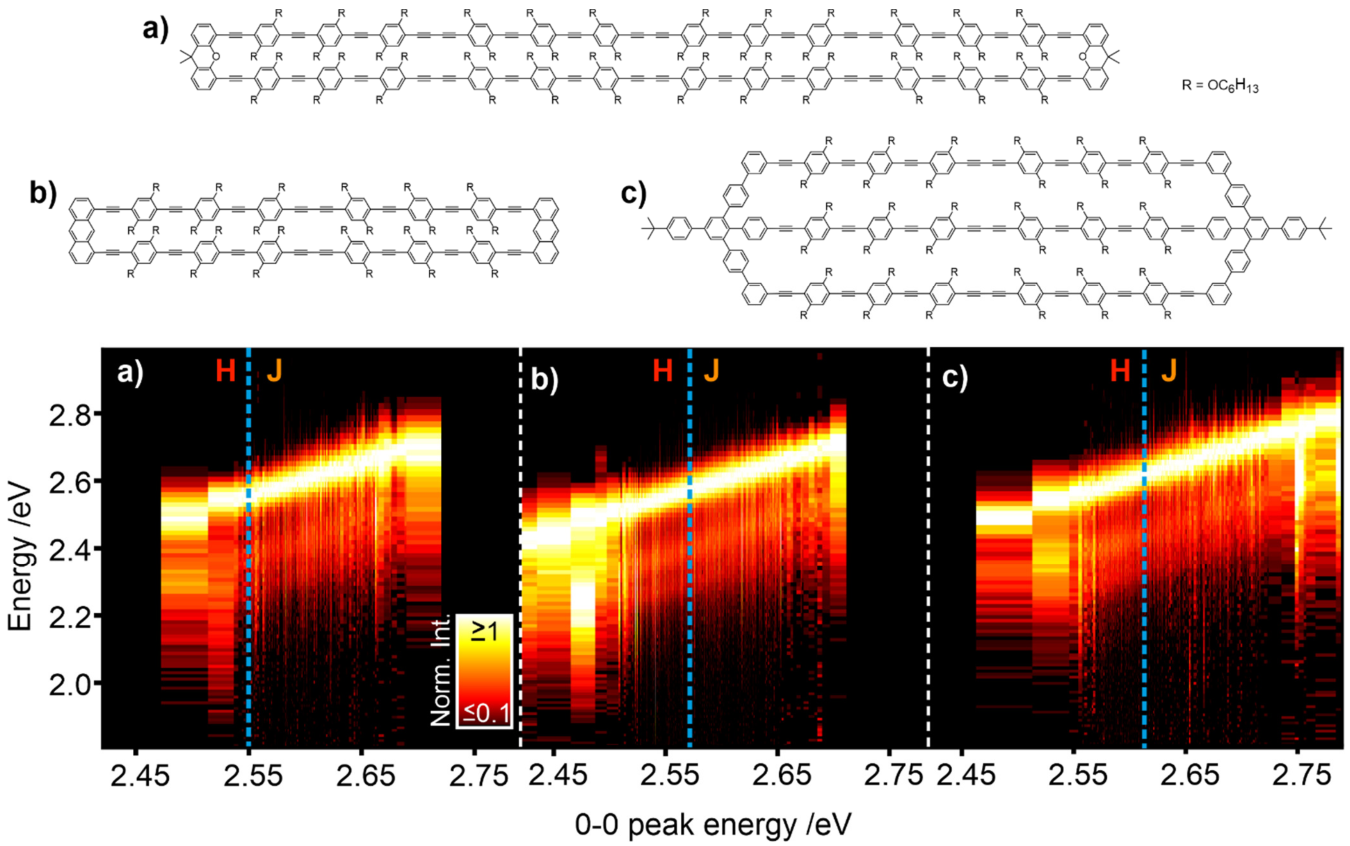

Figure S17. PL spectra of 231, 928 and 484 single molecules with the structures shown on top in panels a), b) and c), sorted by 0-0 peak transition energy $E_{0\text{-}0}$ and normalized to $I_{0\text{-}0}$. Three additional compounds are introduced to substantiate the measurements presented in the main text. The long 12-ring dimer a) is similar to structure **2** in the main text and also shows an initial decrease of the vibronic sideband with increasing $E_{0\text{-}0}$ due to decreased H-type coupling, followed by a subsequent increase as the J-character of the aggregate diminishes. Both H- and J-type coupling are therefore clearly present. b) Another short 6-ring dimer, which behaves analogously to structure **3** shown in the main text. Here, J-type coupling is somewhat less pronounced as compared to the long dimers in a), but the overall trends are apparent. c) Spectra of a 6-ring trimer which also demonstrates signatures of J-type coupling for high peak energies as well as H-type coupling for lower $E_{0\text{-}0}$. The transition region between the dominance of H-type aggregation and that of J-type aggregation is indicated by a dashed blue line.

References

[1] Jester, S.-S.; Schmitz, D.; Eberhagen, F.; Höger, S. *Chem. Commun*. **2011**, *47*, 8838-8840.

[2] Gaefke, G.; Höger, S. *Synthesis* **2008**, 2165-2157.

[3] Takase, M.; Inouye, M. *J. Org. Chem.* **2003**, *68*, 1134-1137.

[4] Eder, T.; Stangl, T.; Gmelch, M.; Remmerssen, K.; Laux, D.; Höger, S.; Lupton, J. M.; Vogelsang, J.; *Nat. Commun.* **2017,** 8, 1641.

[5] Allolio, C.; Stangl, T.; Eder, T.; Schmitz, D.; Vogelsang, J.; Höger, S.; Horinek, D.; Lupton, J. M. *J. Phys. Chem. B* **2018**, *122*, 6431-6441.

[6] McWilliams, K.; Kelly, J. W.; J. *Org. Chem.* **1996**, 61, 7408-7414.

[7] Weston, K. D.; Dyck, M.; Tinnefeld, P.; Müller, C.; Herten D. P.; Dauer, M. *Anal. Chem.* **2002,** 74(20), 5342-5349.

[8] Spano, F. C.; Silva, C. *Annu. Rev. Phys. Chem.* **2014,** 65, 477-500.

[9] Kasha, M. *Radiation Research* **1963,** 20, 55-70.

[10] Chaudhuri, D.; Li, D.; Che, Y.; Shafran, E.; Gerton, J. M.; Zang, L.; Lupton, J. M. *Nano Lett.* **2011,** 11, 488-492.

[11] Stangl, T.; Wilhelm, P.; Schmitz, D.; Remmerssen, K.; Henzel, S.; Jester, S-S.; Höger, S.; Vogelsang, J.; Lupton, J. M.; *J. Phys. Chem. Lett.* **2015,** 6, 1321.

[12] Beljonne, D.; Wittmann, H.F.; Köhler, A.; Graham, S.; Younus, M.; Lewis, J.; Raithby, P.R.; Khan, M.S.; Friend, R.H.; Brédas, J. L.; *J. Chem. Phys.* **1996**, 105, 3868.
